# Maternal Diet During Pregnancy and Offspring Health: Current Evidence on Dietary Patterns, Microbiota, and Developmental Programming

**DOI:** 10.3390/nu18162710

**Published:** 2026-08-19

**Authors:** Dana-Teodora Anton-Păduraru, Dana Elena Mîndru, Codruta Olimpiada Iliescu Haliţchi, Ana Simona Bocec, Olivia Simona Dorneanu, Felicia Trofin, Mioara Florentina Trandafirescu, Lucia Maria Sur, Cătălina Elena Mărgineanu, Paula Popovici, Cosmin Diaconescu, Alina Costina Luca

**Affiliations:** 1 Department of Mother and Child Medicine, “Grigore T. Popa” University of Medicine and Pharmacy, 700115 Iasi, Romania; dana.anton@umfiasi.ro (D.-T.A.-P.); olimpiada.iliescu@umfiasi.ro (C.O.I.H.); ana-simona.drochioi@umfiasi.ro (A.S.B.); paula.popovici@umfiasi.ro (P.P.); diaconescu.cosmin@email.umfiasi.ro (C.D.); alina.luca@umfiasi.ro (A.C.L.); 2 “Sf. Maria” Children Emergency Hospital, 700309 Iasi, Romania; mio.trandafirescu@umfiasi.ro; 3 Microbiology Department, “Grigore T. Popa” University of Medicine and Pharmacy, 700115 Iasi, Romania; olivia.dorneanu@umfiasi.ro (O.S.D.); felicia.trofin@umfiasi.ro (F.T.); 4 Clinical Hospital of Infectious Diseases “Sf. Parascheva”, 700116 Iasi, Romania; 5 “Sf. Spiridon” County Clinical Emergency Hospital, 700111 Iasi, Romania; 6 Department of Morphofunctional Sciences I, “Grigore T. Popa” University of Medicine and Pharmacy, 700115 Iasi, Romania; 7 Department of Child and Mother, “Iuliu Hatieganu” University of Medicine and Pharmacy, 400347 Cluj-Napoca, Romania; sur.maria@umfcluj.ro (L.M.S.); catalina.colt@elearn.umfcluj.ro (C.E.M.); 8 Emergency Children’s Hospital, 400370 Cluj-Napoca, Romania

**Keywords:** pregnancy, offspring, maternal nutrition, microbiota, dietary patterns, gestational diabetes, birth outcomes, offspring health

## Abstract

**Background/Objectives:** Maternal nutrition is an important exogenous factor which, together with other factors, may play a fundamental role in ensuring the well-being of the pregnant woman and in the development of the offspring, with long-term effects on health. Therefore, this narrative review aims to synthesize current evidence on how maternal dietary patterns and nutrient intake during pregnancy affect maternal well-being, pregnancy outcomes, fetal growth, and the short- and long-term health of the child, while addressing research gaps, and implications for antenatal nutrition guidance. Literature Search Strategy and Study Selection: A comprehensive literature search was performed using the electronic databases PubMed and Google Scholar. The selection and curation of articles were guided by the central research question: “How does maternal diet during pregnancy influence maternal health and offspring outcomes?” **Results:** The analysis of the literature reveals that the overall maternal dietary pattern is more important than the isolated intake of nutrients in determining the course of pregnancy and the long-term health of the offspring. **Conclusions:** The present review pointed out that maternal nutrition is not merely energy support for pregnancy, but a factor in fetal programming with a long-term impact on the child, in line with the Developmental Origins of Health and Disease (DOHaD) concept, and may act as an epigenetic and metabolic influence with potential transgenerational effects. Optimizing the diet in the periconceptional period and during pregnancy represents an accessible and effective form of preventive intervention, but it requires personalized recommendations and structured nutritional education begun as early as the preconception period.

## 1. Introduction

According to the DOHaD concept of nutritional programming introduced by David Barker in the 1990s, maternal nutrition during the first 1000 days of life can drive metabolic changes and changes in various organs of the offspring and is considered the most important determinant of fetal intrauterine programming [1]. Maternal nutrition is an important exogenous factor which, together with other factors such as epigenetic modifications, lifestyle, and the intrauterine environment, may play a fundamental role in ensuring the well-being of the pregnant woman and the development of the offspring, with long-term effects on human health [2]. Both maternal overnutrition and undernutrition cause changes in the gut microbiota (GM) and may increase the risk of metabolic and neurological diseases, as well as life-threatening events, in the offspring [3]. Maternal nutrition associated with a low maternal nutritional status (underweight) can affect the offspring (Intrauterine Growth Restriction—IUGR, or Small for Gestational Age—SGA), the latter carrying a risk of developing various conditions in adulthood (obesity, dyslipidemias, type 2 diabetes, renal failure, and cardiovascular disease). During the first two trimesters of pregnancy, maternal undernutrition can increase the prevalence of obesity in the offspring, the explanation being a predictive adaptive response secondary to nutrient intake [4].

Motivation and aim: Maternal diet during pregnancy is a key modifiable factor that influences both maternal health and offspring outcomes. Adequate nutrition supports the physiological demands of pregnancy and may reduce the risk of complications such as excessive gestational weight gain, gestational diabetes, hypertensive disorders, preterm birth, and abnormal fetal growth. At the same time, poor dietary quality, nutrient deficiencies, or overnutrition can affect placental function, fetal development, and long-term offspring health through mechanisms such as metabolic adaptation, inflammation, and fetal programming. Therefore, this narrative review aims to synthesize current evidence on how maternal dietary patterns and nutrient intake during pregnancy affect maternal well-being, pregnancy outcomes, fetal growth, and the short- and long-term health of the child, while identifying research gaps and implications for antenatal nutrition guidance.

## 2. Literature Search Strategy and Study Selection

This narrative review was based on a structured search of PubMed and Google Scholar for English, French and Romanian-language articles published between 2022 and 2026. The search was last updated in July 2026. The main search focused on publications from the preceding five years, although earlier seminal studies were included when they provided essential background or original mechanistic evidence. But although additional databases such as Scopus/Web of Science/Embase are available, PubMed and Google Scholar were considered appropriate for this narrative review because they encompass the vast majority of clinically relevant medical literature and allow comprehensive identification of the principal studies addressing the review topic. Given the narrative nature of this review, these databases were considered sufficient to capture the key evidence relevant to the topic.

Search terms included combinations of “pregnancy,” “offspring,” “maternal nutrition,” “maternal diet,” “microbiota,” “microbiome,” “dietary patterns,” “gestational diabetes,” “hypertensive disorders of pregnancy,” “birth outcomes,” and “offspring health,” combined using “AND” and “OR.” Reference lists of relevant articles were also screened manually. Approximately 200 records were identified, 167 titles and abstracts were screened, and 128 publications were included. Studies were selected according to their relevance to maternal diet, maternal health, placental or fetal development, birth outcomes, the maternal or infant microbiome, and offspring health. Original human studies were prioritized, while systematic reviews and meta-analyses were used to summarize the wider evidence and identify primary studies. Animal and in vitro studies were included mainly to explain potential mechanisms and were distinguished from human evidence. This review focuses on offspring outcomes for which there is emerging evidence linking maternal diet, maternal microbiota, and immune–metabolic programming within a DOHaD framework. We focus on obesity and metabolic disease, allergic disease, neurodevelopmental outcomes, and cardiovascular risk. Other microbiota-related conditions such as autoimmune diseases, inflammatory bowel disease, and cancer were not included due to limited pregnancy-specific data or because their primary developmental windows occur later in life and are less directly linked to antenatal nutrition.

Conflicting findings were retained and discussed, with greater weight given to prospective or controlled studies, larger samples, appropriate adjustment for confounding factors, and findings replicated across independent populations. The extent of coverage for each topic reflected the quantity, quality, and direct relevance of the available pregnancy-specific evidence. Generative artificial intelligence was not used to search for, select, evaluate, or synthesize the literature. It was used only to generate figures, which were reviewed and approved by the authors.

## 3. Results

### 3.1. Diet–Microbiota Interactions in Pregnancy

The human microbiota comprises communities of microorganisms, including bacteria, archaea, fungi, and viruses, which inhabit different body sites and contribute to metabolic and immunological homeostasis. Pregnancy is accompanied by substantial hormonal, metabolic, and immune adaptations that may influence the composition, diversity, and relative abundance of the maternal gut microbiota from the first to the third trimester, as suggested by both human and animal studies [2,5,6]. However, findings from longitudinal human studies remain inconsistent. Some studies have reported increased *Pseudomonadota* and *Actinomycetota*, reduced *Faecalibacterium*, and lower alpha-diversity in late pregnancy, whereas others have observed relative stability of the gut microbiota throughout pregnancy [7]. Differences in study populations, prepregnancy body mass index, dietary intake, medication and antibiotic exposure, geographic setting, sampling time points, sequencing methods, and statistical analyses may partly account for these discrepancies. Therefore, pregnancy-related remodeling of the maternal gut microbiota should not be considered universal but rather dependent on maternal, environmental, and methodological factors.

The maternal gut microbiota may nevertheless influence fetal development and subsequent offspring immunity, metabolism, brain function, and behavior through microbial metabolites, immune mediators, and placental signaling. Although microbial DNA has been detected in placental tissue, amniotic fluid, and meconium in some studies, whether a healthy fetus harbors a stable resident microbiota remains unresolved. The interpretation of findings from these low-biomass samples is complicated by contamination introduced during sample collection, DNA extraction, sequencing, and delivery. Accordingly, direct transplacental transmission of microorganisms should be regarded as a hypothesis rather than an established physiological mechanism. Maternal dysbiosis associated with high-fat diets, infections, antibiotic exposure, or stress has been linked to adverse immunological, gastrointestinal, metabolic, neurobehavioral, obesity-related, and hypertensive outcomes in offspring; however, causality has not been firmly established in humans [1,6,7].

Maternal diet is an important modifiable determinant of the maternal gut microbiota and may also influence the human milk and infant gut microbiomes [6,8,9,10,11]. Plant foods, including whole grains, legumes, fruits, vegetables, nuts, and seeds, provide microbiota-accessible carbohydrates, dietary fiber, polyphenols, and other bioactive compounds that can support saccharolytic microorganisms and microbial production of short-chain fatty acids (SCFAs), particularly acetate, propionate, and butyrate. These metabolites contribute to intestinal barrier integrity, immune regulation, and metabolic signaling and may mediate communication between the maternal gut microbiota, placenta, and developing fetus without requiring direct fetal microbial colonization.

Early intestinal colonization accelerates during and after birth and is strongly influenced by delivery mode, gestational age, breastfeeding, antibiotic exposure, the surrounding environment, and contact with maternal microbial communities. The neonatal gut microbiota is less diverse than the adult microbiota and is frequently enriched in members of *Actinomycetota* and *Pseudomonadota*. Nevertheless, associations between maternal diet and maternal or infant microbial composition vary according to the type and amount of fiber consumed, the overall dietary pattern, baseline microbiota, maternal metabolic status, gestational stage, geographic location, and delivery mode [6,8,9,10,11,12,13,14,15,16,17]. Current evidence therefore supports a role for maternal dietary composition, particularly plant foods rich in fiber and bioactive compounds, in modulating maternal and early-life microbial ecosystems, while evidence for a distinct resident fetal microbiome remains insufficient.

A favorable gut microbial ecosystem is characterized not simply by the presence of particular bacteria, but by functional properties such as the fermentation of microbiota-accessible carbohydrates, production of SCFAs, maintenance of colonization resistance, preservation of intestinal barrier integrity, vitamin biosynthesis, and regulation of mucosal and systemic immune responses. Butyrate is particularly relevant because it is a major energy source for colonocytes, supports epithelial hypoxia and tight-junction integrity, and contributes to immune tolerance, including the differentiation and activity of regulatory T cells. By maintaining epithelial homeostasis and limiting luminal oxygen availability, butyrate-producing communities may also restrict the expansion of facultative anaerobes and opportunistic pathogens [18,19].

Several members of the families *Lachnospiraceae* and *Ruminococcaceae*, including species belonging to *Faecalibacterium*, *Roseburia*, *Anaerostipes*, *Eubacterium*, and *Coprococcus*, are recognized as important butyrate producers. However, taxonomic abundance alone does not necessarily reflect butyrate production because metabolic activity depends on substrate availability, strain-level characteristics, microbial cross-feeding, and the overall intestinal environment. In addition, different members of the gut microbiota can synthesize B-group vitamins and menaquinones collectively referred to as vitamin K2, although the amount available to the host and its physiological significance vary among vitamins, microbial species, and intestinal sites [18,20]. These vitamin-producing organisms should therefore not be considered synonymous with butyrate-producing bacteria.

Conversely, potentially unfavorable microbial patterns include reduced SCFA-producing capacity, impaired colonization resistance, increased intestinal permeability, and expansion of inflammation-associated pathobionts. Increased abundance of facultative anaerobic bacteria, including some members of the *Enterobacteriaceae*, may occur in an inflamed and oxygen-enriched intestinal environment. Their products, including lipopolysaccharide, can activate innate immune pathways and may contribute to local and systemic low-grade inflammation when epithelial barrier function is impaired. Nevertheless, individual taxa should not be classified as universally beneficial or harmful because their effects depend on species and strain, relative abundance, community context, host characteristics, and metabolic activity [19,21].

Dietary components can promote or impair these microbial functions. Dietary fiber, resistant starch, inulin, oligosaccharides, and other microbiota-accessible carbohydrates provide substrates for microbial fermentation and cross-feeding, thereby supporting SCFA-producing communities. Plant foods also provide polyphenols and other bioactive compounds that may influence microbial composition, intestinal barrier function, and inflammatory signaling. In contrast, diets low in fermentable fiber and high in saturated fat, refined carbohydrates, or ultra-processed foods may reduce SCFAs production, impair barrier integrity, and favor pro-inflammatory microbial and metabolic profiles. The taxonomic changes described below should therefore be interpreted in relation to these functional outcomes rather than as isolated increases or decreases in individual bacterial groups or pathogens [18,19].

**Figure 1:**
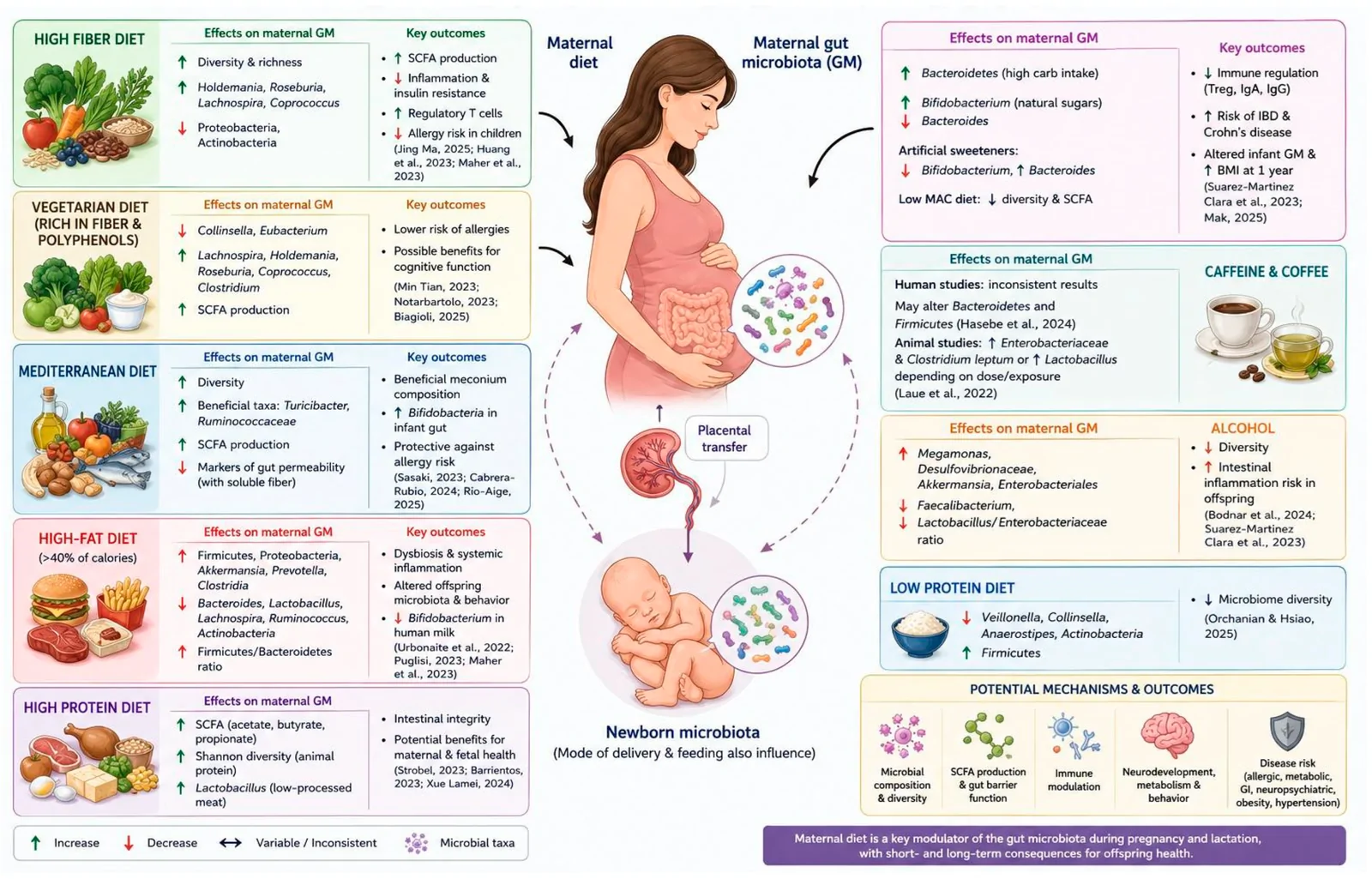
The impact of maternal diet on the microbiota [2,8,9,10,11,12,13,15,17,22,25,26,27,28,29,30,31].

The evidence discussed in the following subsections derives from heterogeneous study designs, including observational studies in pregnant women, mother–infant cohorts, animal experiments, and, in some cases, in vitro or nonpregnancy studies. These sources of evidence are not directly comparable. Human pregnancy studies are therefore prioritized when assessing clinical relevance, whereas animal and in vitro studies are used primarily to support biological plausibility. Observational associations should not be interpreted as causal, and findings reported in a single cohort are considered preliminary until replicated independently. Differences in study population, maternal body mass index and metabolic status, gestational timing, dietary assessment, antibiotic exposure, specimen type, sequencing methods, and statistical analysis may partly explain contradictory taxonomic findings [7,11,12,13,22,23,24,25,26]. Accordingly, the following discussion emphasizes functional outcomes—including microbial fermentation, short-chain fatty acid production, intestinal barrier integrity, immune regulation, and inflammatory signaling—while detailed changes in individual taxa are interpreted cautiously (Figure 1).

#### 3.1.1. Dietary Fiber and Influence on GM

Dietary fiber is an important determinant of microbial fermentation during pregnancy. Fermentable fibers and resistant starches support microbial cross-feeding and the production of SCFAs, particularly butyrate, by members of *Lachnospiraceae* and *Ruminococcaceae*. Therefore, associations between fiber intake and individual bacterial taxa should be considered together with functional outcomes such as SCFAs production, microbial diversity, intestinal barrier integrity, and inflammatory status [22].

Low-fiber diets have been associated with reduced abundance of *Bacteroides*, *Veillonella*, *Paraprevotella*, and with increased abundance of *Firmicutes*, *Sutterella*, *Ruminococcus*, and *Collinsella* [22]. Diets low in fruits and vegetables have also been linked to higher abundance of *Prevotella*, *Clostridia*, *Lachnospira*, *Hungatella*, *Ruminococcaceae*, *Megamonas*, *Sutterella*, and *Burkholderiaceae* [11]. However, many of these taxon-level findings have not been consistently replicated and should be interpreted in light of differences in study design, dietary assessment, gestational stage, maternal metabolic status, and microbiome-analysis methods.

In contrast, higher fiber intake has been associated with greater gut microbial richness and diversity, including higher abundance of *Holdemania*, *Roseburia*, *Lachnospira*, and *Coprococcus* and lower abundance of *Proteobacteria* and *Actinobacteria* [12]. Experimental evidence also suggests that fermentable fiber may improve maternal metabolic and inflammatory outcomes. In a mouse model, Huang et al. (2023) [27] found that a high-fiber diet reduced insulin resistance (IR) and placental inflammation, increased *Lachnospiraceae* abundance, and limited the transfer of bacterial lipopolysaccharide. These effects were accompanied by increased SCFAs production, higher interleukin-10 (IL-10) and immunoglobulin G (IgG) levels, lower interferon-gamma (IFN-γ) levels, reduced inflammation, and improved antioxidant capacity. Higher fiber intake has also been positively associated with gut microbial diversity and abundance in overweight pregnant women [28]. In another experimental study, Liu et al. (2021) reported that maternal fiber intake improved microbiome composition and reduced synaptic damage and abnormalities in microglial maturation in offspring [32]. These neurodevelopmental findings are promising but remain to be confirmed in human studies.

Plant-based and vegetarian diets are generally rich in fiber, polyphenols, antioxidants, and other phytochemicals and may therefore influence gut microbial composition. During early pregnancy, vegetarian diets have been associated with lower beta-diversity, reduced abundance of *Collinsella* and *Eubacterium*, and increased abundance of *Lachnospira*, *Holdemania*, *Roseburia*, *Coprococcus*, and *Clostridium*, several of which are involved in SCFAs production [22,26]. Vegetarian mothers have also been reported to have lower levels of *Collinsella*, *Holdemania*, and *Eubacterium* and higher levels of *Roseburia* and *Lachnospiraceae* [11]. Diets rich in plant foods, polyphenols, and yogurt have additionally been associated with a lower risk of allergic outcomes [22,26].

Evidence regarding the effects of maternal plant-food intake on the neonatal microbiome remains limited and inconsistent. Lundgren et al. (2018) reported an inverse association between maternal fruit and vegetable intake and *Bifidobacterium* abundance in vaginally delivered infants [33]. By contrast, Fan et al. (2021) found that high or low maternal fruit and vegetable intake did not alter maternal alpha-diversity, although higher intake was associated with greater abundance of *Propionibacterium*, *Tannerellaceae*, *Lactococcus*, *Parabacteroides*, and *Cutibacterium* in infants [34]. These differences indicate that associations between maternal diet and the infant microbiome may depend on factors such as delivery mode, study population, and the timing of microbiome assessment. The relationship between vegetarian diets and telomere length is also inconsistent, possibly because of differences in overall diet quality, nutrient bioavailability, and the consumption of refined plant-based foods [35].

The Mediterranean diet is another plant-rich dietary pattern that may favorably influence the gut microbiome during pregnancy. Its main components—including whole-grain cereals, fruits, vegetables, legumes, nuts, fiber, polyphenols, and unsaturated fatty acids—have been associated with greater microbial diversity [14]. Experimental findings suggest that this dietary pattern may also influence cecal SCFAs production and increase taxa such as *Turicibacter* and *Ruminococcaceae* [15]. Soluble fiber may further support gut microbial composition, reduce markers of intestinal permeability, and increase metabolites involved in immune tolerance [16].

Associations between maternal adherence to a Mediterranean-type diet and the infant microbiome may be modified by delivery mode. Maher et al. (2023) found positive associations with *Enterobacteriaceae* and *Streptococcus* in vaginally delivered infants but inverse associations with the same taxa in infants delivered by cesarean section [12]. Fan et al. (2021) also reported that maternal consumption of fruits and vegetables rich in fiber, fructose, folate, and ascorbic acid was inversely associated with *Lachnospiraceae*, *Proteobacteria*, and *Erysipelatoclostridium* [34].

Greater adherence to the Mediterranean diet has additionally been associated with higher levels of *Firmicutes* and *Bacteroidetes* in neonatal meconium [10]. In the MAMI study, a maternal diet rich in vegetables and yogurt was associated with greater *Bifidobacterium* abundance in the infant gut and may have contributed to a lower risk of allergic outcomes [17].

The benefits associated with the Mediterranean diet should not be attributed to a low total fat intake. Traditional Mediterranean diets may provide a relatively high proportion of energy from fat, but most of this fat comes from olive oil, nuts, seeds, and fish and is consumed within a dietary pattern rich in fiber, polyphenols, and other plant-derived bioactive compounds. Thus, fatty-acid quality and food source may be more relevant to diet–microbiota relationships than the percentage of energy derived from fat alone.

Overall, current evidence more consistently supports an association with fiber-rich dietary patterns, microbial fermentation, SCFAs production, and intestinal or immune homeostasis than with changes in any single bacterial taxon. Many taxonomic findings remain observational, study-specific, or dependent on delivery mode and therefore require confirmation in larger, well-controlled pregnancy cohorts (Figure 1).

#### 3.1.2. Dietary Fat Quality, Quantity, and Microbial Diversity

The relationship between maternal fat intake and the GM depends not only on the amount of fat consumed but also on its fatty-acid composition, food source, accompanying nutrients, and the overall dietary pattern. In experimental studies, a high-fat diet is commonly defined as one providing more than 40% of total energy from fat. These diets are often rich in saturated fat, energy dense, low in fermentable fiber, and sometimes combined with refined carbohydrates or sucrose.

The traditional Med diet in which approximately 42–43% of total energy intake came from fat, but olive oil was the main source, illustrates this distinction. The diet was consequently rich in monounsaturated oleic acid and relatively low in saturated fat. It also included vegetables, legumes, whole grains, fruits, and nuts, providing substantial amounts of fiber, polyphenols, and other bioactive compounds. A Med diet with a relatively high fat content is therefore not equivalent to a Western-style diet rich in saturated fat and poor in plant foods.

Most evidence on maternal high-fat diets comes from animal studies. In these models, maternal high-fat intake has been associated with microbial dysbiosis and altered microbial colonization in offspring, with some changes persisting for at least 6 weeks after birth [8]. Reported maternal microbial changes include higher abundance of *Prevotella*, *Akkermansia*, *Methanobrevibacter*, *Romboutsia*, *Clostridia, Proteobacteria*, *Verrucomicrobia*, *Tenericutes* and *Firmicutes*, together with lower abundance of *Bacteroides*, *Lactobacillus*, *Lachnospira*, *Ruminococcus*, *Actinobacteria,* and *Treponema*. An increased *Firmicutes*-to-*Bacteroidetes* ratio has also been reported, along with bacterial overgrowth, systemic inflammation, and immune activation [24,28].

Maternal high-fat intake has also been associated with lower levels of *Bifidobacterium* and polyamines in human milk [28]. In experimental models, some of these alterations were partially reversed by prebiotic supplementation, which increased bifidobacterial abundance and supported recovery of the microbial community [3,7,8,13,18,22].

Other experimental findings suggest that maternal high-fat intake may influence the hypothalamic–pituitary–adrenal axis, with possible consequences for fetal neurodevelopment and depressive-like behavior later in life [3]. High-fat feeding may also favor the expansion of *Bilophila wadsworthia*, a bile-tolerant bacterium associated with intestinal inflammation. In animal models, diets rich in both fat and sucrose have been associated with lower *Bifidobacterium* and higher *Clostridium* abundance [2,9]. These observations provide mechanistic support but cannot be assumed to reflect the effects of habitual fat intake in pregnant women.

Fatty-acid composition may partly explain the variation between studies [12,13]. Saturated-fat intake has been associated with the expression of pro-inflammatory genes and with changes in microbial diversity. When saturated and monounsaturated fatty acids were considered together, higher intake was also associated with greater *Firmicutes* abundance [2,17,26]. During the second trimester, diets rich in monounsaturated fatty acids, cholesterol, and vitamin D have been linked to higher *Proteobacteria* abundance, whereas cholesterol intake and dietary factors related to SCFAs have been associated with differences in alpha-diversity [12,13,18,22]. These findings are mainly observational and have not been consistently reproduced.

Monounsaturated and polyunsaturated fatty acids may have different effects from saturated fat. Their intake has been associated with greater bacterial diversity and increased abundance of *Ruminococcus* and *Paraprevotella* during pregnancy [11]. Higher polyunsaturated fatty-acid intake, particularly linoleic acid, has also been associated with *Holdemania*, *Roseburia*, and *Collinsella* [12,13]. Omega-3 fatty acids may influence placental inflammatory signaling and have been linked to higher abundance of *Ruminococcaceae*, *Lachnospira*, *Blautia*, *Coprococcus*, *Bifidobacterium*, and *Oscillospira* [12,22,25,36]. However, the direction and clinical significance of these taxonomic associations remain uncertain.

Food sources of fat may also influence maternal and infant microbial composition. Lipids consumed as part of a Mediterranean dietary pattern have been associated with lower *Bacteroidetes* and higher *Firmicutes* abundance [11]. Maternal fish intake has been positively associated with *Streptococcus* in infants delivered vaginally or by cesarean section, whereas red-meat intake has been positively associated with *Bifidobacterium* in infants delivered by cesarean section [12,13]. Fish-oil intake has been linked to higher *Bacteroidetes*, *Enterococcus faecium*, and *Bilophila wadsworthia* and to lower *Enterobacteriaceae* abundance [13,18]. These findings indicate that delivery mode and food source may modify associations between dietary fat and the infant microbiome.

Fat-soluble vitamin intake may provide another source of variation. Diets rich in fat-soluble vitamins have been associated with lower microbial alpha-diversity. Higher vitamin D intake, in particular, has been linked to reduced alpha-diversity and greater *Proteobacteria* and *Actinobacteria* abundance [12,22]. These associations should be interpreted cautiously, as they do not establish that vitamin intake directly causes dysbiosis or subsequent disease.

Taken together, the evidence suggests that the microbiome effects of dietary fat cannot be predicted from total fat intake alone. Diets rich in saturated fat, refined carbohydrates, and ultra-processed foods differ markedly from dietary patterns in which fat is derived mainly from olive oil, nuts, seeds, and fish and is consumed alongside fiber- and polyphenol-rich foods. Both fat quantity and quality may be relevant, but pregnancy-specific human evidence remains limited, and many reported taxonomic changes require confirmation in independent cohorts (Figure 1).

#### 3.1.3. Proteins and Influence on GM

Evidence on protein intake and the GM during pregnancy remains limited, and the reported associations appear to depend on both the amount and source of protein. High-protein diets have been associated with higher concentrations of acetate, butyrate, and propionate [11,29]. However, these SCFAs are produced mainly through microbial fermentation of carbohydrates, and their concentrations may therefore reflect the availability of fermentable substrates and microbial cross-feeding, rather than protein intake alone.

In observational studies, animal-protein intake during pregnancy has been positively correlated with the maternal gut Shannon diversity index, while minimally processed meat consumption has been associated with greater *Lactobacillus* abundance [11]. Diets containing plant proteins and polyphenols have been linked to higher abundance of *Eubacterium*, *Dehalobacterium*, and *Christensenellaceae*. By contrast, high-protein, low-carbohydrate diets have been associated with lower *Roseburia* abundance, possibly because reduced carbohydrate availability limits the growth of bacteria involved in fiber fermentation [22,26].

Low-protein diets may also affect the maternal microbiota. They have been associated with reduced microbial diversity, lower abundance of *Veillonella*, *Collinsella*, *Anaerostipes*, and *Actinobacteria*, and higher *Firmicutes* abundance [30]. Nevertheless, these findings have not been replicated sufficiently to establish a consistent pregnancy-specific microbial profile. The effects of protein intake are likely to vary with protein source, degree of food processing, carbohydrate and fiber intake, maternal metabolic status, and the composition of the baseline microbiota (Figure 1).

#### 3.1.4. High-Sugar Diets, Sweeteners, and Microbial Diversity

The effects of carbohydrates on the maternal and infant microbiota depend on their source, structure, and accessibility to intestinal microorganisms. Higher carbohydrate intake has been associated with increased Bacteroidetes abundance in the human milk microbiota [11]. Natural sugars have been linked to higher *Bifidobacterium* and lower *Bacteroides* abundance, whereas artificial sweeteners have been associated with the opposite pattern [26]. These observations remain preliminary and should not be interpreted as showing that all naturally occurring sugars have beneficial microbial effects.

Oligosaccharides can serve as substrates for commensal microorganisms and support the growth of *Bifidobacterium*, *Lactobacillus*, and *Bacteroides* [37]. More broadly, microbiota-accessible carbohydrates support microbial fermentation and SCFAs production. Diets deficient in these carbohydrates may reduce microbial diversity and SCFAs availability, impair regulatory T cells function, and decrease immunoglobulin A (IgA) and IgG production [13].

Artificial sweeteners, emulsifiers, and other food additives have also been linked to changes in microbial composition and inflammatory signaling. Reported changes include increased *Bacteroides* and reduced *Firmicutes* abundance, together with impaired barrier function and a microbial environment associated with intestinal inflammation. Ultra-processed food consumption has similarly been associated with a higher risk of Crohn’s disease [9]. However, much of this evidence comes from animal models, in vitro studies, or nonpregnant populations and should therefore be regarded as mechanistic rather than pregnancy-specific evidence.

In a mother–infant cohort, Laforest-Lapoint et al. (2021) found that maternal consumption of artificially sweetened beverages during pregnancy was associated with differences in the infant GM at 3–4 and 12 months of age, including lower *Bacteroides* abundance [38]. Maternal intake was also associated with a higher infant body mass index at 1 year [13]. Because this was an observational association, confounding by maternal adiposity, metabolic health, overall dietary quality, and lifestyle factors cannot be excluded.

Current evidence therefore supports a distinction between fermentable carbohydrates, which provide substrates for microbial metabolism, and refined sugars or nonnutritive sweeteners, which may have different effects. Nevertheless, pregnancy-specific data remain sparse, and the effects of individual sweeteners or carbohydrate sources require confirmation in controlled human studies (Figure 1).

#### 3.1.5. Caffeine, Alcohol, Tea, and Impact on GM

Evidence on prenatal caffeine exposure and the GM is limited and inconsistent. Struniewicz et al. (2025) found no significant association between prenatal caffeine exposure and microbial alpha-diversity, the relative abundance of bacterial species or phyla, or predicted functional pathways [39]. In contrast, Hasebe et al. (2024) reported that caffeine consumption during pregnancy may alter the abundance of *Bacteroidetes* and *Firmicutes* and affect microbial diversity in breastfed infants [40]. Differences in caffeine dose, source, exposure assessment, biological sample, and timing of infant microbiome analysis may explain the conflicting findings.

Experimental studies also suggest that coffee and caffeine can influence gut microbial composition, although the results vary according to the preparation and dose. In rats given caffeinated coffee with standard chow, Laue et al. (2022) observed higher *Enterobacteriaceae* and *Clostridium leptum* abundance [41]. Because coffee contains polyphenols and other bioactive compounds, these effects cannot be attributed to caffeine alone. A separate experiment reported higher *Lactobacillus* abundance in rats exposed repeatedly to high doses of caffeine than in untreated animals. These animal findings provide mechanistic information but cannot be directly extrapolated to caffeine consumption during human pregnancy.

Prenatal alcohol exposure appears to have more consistently adverse associations with the offspring microbiota. It has been linked to higher *Megamonas* and lower *Faecalibacterium* abundance, the latter being a genus associated with anti-inflammatory activity [18]. In an animal study, Bodnar et al. (2024) found that alcohol exposure during pregnancy increased aerotolerant and inflammation-associated microorganisms, including *Desulfovibrionaceae*, *Akkermansia*, and members of *Enterobacterales* [31]. The *Lactobacillus*-to-*Enterobacteriaceae* ratio was also lower in alcohol-exposed dams and their offspring. Although these findings support a biological effect of prenatal alcohol exposure, human taxon-level evidence remains limited.

Tea contains polyphenols, particularly flavonoids, as well as alkaloids, amino acids, polysaccharides, proteins, and minerals. Experimental and nonpregnancy studies suggest that tea polyphenols may support the growth of *Bifidobacteriaceae* and *Lactobacillaceae* while inhibiting organisms such as *Clostridioides difficile*, *Clostridium perfringens*, *Escherichia coli*, *Helicobacter pylori*, and some members of *Prevotella*. Tea polysaccharides may act as fermentable, prebiotic-like substrates, contribute to SCFAs production, and alter the abundance of *Prevotella* and members of *Bacteroidetes* [42]. These effects should not be described as established during pregnancy because the available evidence is predominantly experimental.

In summary, no consistent pregnancy-specific microbial profile has been identified for caffeine or tea. The evidence concerning alcohol is more consistently unfavorable, particularly in animal models, but the extent to which particular microbial changes mediate alcohol-related fetal or infant outcomes remains uncertain (Figure 1).

#### 3.1.6. Integrated Maternal Diet–Microbiome–Placenta–Offspring Axis

Maternal diet may influence fetal development through an interconnected pathway involving the maternal GM, microbial metabolites, maternal immune and metabolic responses, placental function, and epigenetic regulation. In this framework, dietary components do not act solely as nutrients absorbed by the mother. They also provide substrates for microbial metabolism and can alter the functional capacity of the maternal GM. The resulting metabolites and immune signals may enter the maternal circulation, interact with the placenta, and influence fetal development without requiring direct microbial colonization of the placenta or fetus [43,44,45].

Fiber-rich foods provide fermentable carbohydrates that support microbial cross-feeding and the production of SCFAs, including acetate, propionate, and butyrate. These metabolites contribute to intestinal barrier integrity, immune regulation, glucose and lipid metabolism, and protection against the expansion of facultative anaerobes and opportunistic pathogens [43,44]. In contrast, diets low in fiber and rich in saturated fat or refined carbohydrates may reduce SCFAs production, increase intestinal permeability, and facilitate the passage of microbial products such as lipopolysaccharide into the maternal circulation. This may promote low-grade systemic inflammation and alter the metabolic environment to which the placenta is exposed [43,46].

Other microbial metabolites may also participate in maternal–placental communication. Gut bacteria modify primary bile acids into secondary bile acids, which can signal through receptors involved in glucose metabolism, lipid metabolism, inflammation, and vascular function. Microbial metabolism of tryptophan produces indole derivatives that may influence intestinal barrier function and immune tolerance through aryl hydrocarbon receptor signaling. In addition, the microbiota contributes to the metabolism or synthesis of folate, choline-related compounds, and several B-group vitamins involved in one-carbon metabolism. However, evidence directly linking these pathways to pregnancy outcomes in humans remains limited, and much of the mechanistic evidence comes from animal or in vitro studies [47,48,49].

The placenta is likely to function as an important intermediary in this pathway rather than as a passive barrier. Changes in maternal circulating metabolites, inflammatory mediators, hormones, and nutrients may influence trophoblast function, placental vascular development, oxidative stress, nutrient transport, and endocrine signaling. SCFAs may affect placental metabolism and immune regulation through G-protein-coupled receptors and other signaling pathways, whereas increased exposure to lipopolysaccharide and pro-inflammatory cytokines may activate Toll-like receptor and nuclear factor-kappa B signaling. Persistent activation of these pathways may contribute to placental inflammation and impaired nutrient exchange, although direct evidence in pregnant women is less extensive than evidence from experimental models [43,46,50].

Experimental studies support the possibility that microbial metabolites participate in fetal metabolic, immune, and neurological programming. In mice, maternal microbiota-derived SCFAs reached the maternal circulation and fetal tissues and signaled through free fatty acid receptors involved in the development of the fetal nervous system, intestinal epithelium, and pancreas. Disruption of this pathway increased the offspring’s later susceptibility to obesity and impaired glucose regulation [44]. Other animal studies have shown that the maternal microbiota influences fetal brain metabolite availability and thalamocortical axon development [45]. Maternal microbial signals may also prepare the neonatal immune system for postnatal microbial exposure by affecting intestinal innate immune cells, epithelial antimicrobial responses, and tolerance to microbial molecules [51]. These findings provide biological plausibility, but equivalent causal pathways have not yet been established in humans.

Epigenetic regulation represents another possible link between maternal diet, microbial activity, placental function, and long-term offspring health. SCFAs, particularly butyrate, can inhibit histone deacetylases and thereby alter histone acetylation and gene transcription. Microbial and dietary contributions to folate, choline, methionine, and B-vitamin metabolism may also affect the availability of methyl donors required for DNA methylation. Through these mechanisms, changes in maternal diet and microbial metabolism could modify the expression of genes involved in placental inflammation, nutrient transport, fetal growth, immune development, and energy metabolism [47,48,49,52]. In human placental tissue, maternal consumption of olive oil and fish has been associated with differences in histone acetylation at immune-regulatory genes, although such observational associations do not demonstrate that the microbiome mediated the effects [53].

The consequences of this interconnected pathway may extend beyond pregnancy. Altered microbial metabolism and placental signaling have been linked experimentally to offspring immune maturation, energy balance, adiposity, glucose regulation, intestinal development, and neurobehavioral outcomes [44,45,50,51]. After birth, these prenatal influences may interact with delivery mode, antibiotic exposure, breastfeeding, human milk composition, complementary feeding, and the developing infant microbiome. Thus, offspring outcomes are unlikely to result from a single maternal dietary component or bacterial taxon but rather from combined changes in microbial community function, metabolite availability, placental responses, and postnatal exposures.

This integrated model should nevertheless be interpreted cautiously. Most human studies are observational and measure maternal diet, microbial composition, circulating metabolites, placental markers, or child outcomes at separate time points. Few studies have assessed all components of the pathway within the same mother–infant cohort. In addition, evidence for epigenetic mediation is stronger in experimental models than in humans. Future studies should combine detailed dietary assessment with longitudinal metagenomics, microbial and host metabolomics, placental transcriptomic and epigenetic analyses, and long-term evaluation of offspring outcomes. Such approaches are needed to determine whether the maternal microbiome is a causal mediator of dietary effects or primarily a marker of broader maternal metabolic and environmental exposures.

### 3.2. Impact of Components of the Diet on Maternal and Offspring Health

During pregnancy, essential changes occur for fetal development that can affect maternal metabolism of various macronutrients and their assimilation, but an adequate diet is also necessary for the development of maternal organs (breasts, uterus, placenta). Increased hepatic glucose production occurs to counteract transient IR and decreased blood glucose—consequences of endocrine-metabolic adaptation. In addition, the diet of the pregnant woman is an epigenetic determinant that can influence the risk of chronic diseases such as cardiovascular diseases and obesity in the offspring [1].

#### 3.2.1. Impact of Lipid Consumption on Maternal and Offspring Health

A high-fat diet contributes to the emergence of changes in weight regulation at the level of the hypothalamus and in energy homeostasis in the fetus, the mechanism being the impairment of the regulation of the expression of the genes encoding the leptin receptor, pro-opiomelanocortin (POMC), and neuropeptide Y, with the consequences being changes in the child’s eating behavior. A high-fat diet during pregnancy also was linked to high circulating insulin levels in the newborn, abnormalities in the endocrine pancreatic morphology of the offspring, and an altered balance of islet endocrine cell development [43]. It also leads to the accumulation of fat mass, impairment of the fetus’s body composition and the activity of pro-inflammatory cytokines, and lipolysis of adipose tissue, which results in the release of free fatty acids in the fetus, negatively influencing glucose tolerance [4,25]. Ceasrine et al. (2022), in a study on the consumption of a high-fat diet by pregnant women, observed that lipid accumulation in utero led to disrupted 5-hydroxytryptamine (5-HT) levels in the fetal brain of males and to the accumulation of triglycerides in the placenta, associated with a pro-inflammatory status in newborns of both sexes [44].

PUFA intake during pregnancy can influence body composition in the offspring, with an elevated omega-3 concentration being associated with a lower body fat percentage, while elevated omega-6 concentrations were correlated with greater preperitoneal abdominal fat and a higher body fat percentage. An increased omega-6/omega-3 ratio is associated with greater abdominal and total fat masses in offspring [1]. Omega-3 fatty acids, especially docosahexaenoic acid (DHA) and eicosapentaenoic acid (EPA), influence lipid metabolism and mitochondrial function, are involved in redox regulation, and reduce the risk of preterm birth [36]. Omega-3 fatty acids, with their anti-inflammatory properties, can ameliorate allergic manifestations, whereas omega-6 fatty acids, with their pro-inflammatory properties, can promote the development of allergies. The European Food Safety Authority (EFSA) recommends for pregnant women an intake of 250 mg/day of EPA and DHA, of which 100–200 mg/day should be DHA [45]. The consumption of foods containing DHA by obese women or women with gestational diabetes (GD) in the last trimester of pregnancy has beneficial effects on adiposity in exclusively breastfed infants [4]. A diet containing SCFAs, by promoting the prolife-ration and differentiation of regulatory T cells (Treg cells), can modulate carbohydrate and lipid metabolism, control insulin levels, and have anti-inflammatory effects [29].

#### 3.2.2. Impact of Carbohydrate Consumption on Maternal and Offspring Health

An increased intake of carbohydrates, especially refined ones, increases weight, leads to large for gestational age (LGA), and increases the risk of diabetes and cardiovascular disease in the pregnant woman. According to the study by Renault et al. (2015), in obese pregnant women, consuming sweets ≥2 times per day at baseline was associated with 5.4 kg greater gestational weight gain compared to <1 time per week, and an 84% higher risk of excessive gestational weight gain [54]. A diet rich in carbohydrates in the second trimester of pregnancy is negatively correlated with the fat index, and in the third trimester with a lower index in the newborn. In the offspring, it raises the risk of obesity and of impaired neurological development, and leads to higher values of cholesterol and triglycerides as well as of blood pressure, while sugar consumption raises insulinemia and promotes the storage of fat in adipose tissue. However, not only the quantity but also the quality of the carbohydrates can have effects on the pregnant woman and the offspring [25]. The consumption of carbohydrates derived from refined cereals, white sugar, and sweetened beverages induces the shortening of TL in cord blood, as does a diet rich in saturated fatty acids and poor in PUFAs [35].

A diet with a low carbohydrate intake has beneficial effects: it improves IR and reduces glycemic values and the risk of GD, and in pregnant women with GD it can improve glycemia. But it can result in low maternal fertility and may contribute to stunted growth in the offspring. However, in the long term it can lead to ketonuria, ketoacidosis, and malnutrition. The consequence of low carbohydrate consumption is the consumption of a greater quantity of fat and protein, in order to compensate for the lack of carbohydrates and to ensure caloric requirements, with effects on the retinal veins, which will have wider diameters and suboptimal microvasculature, as well as IR, impaired glucose tolerance, and decreased mitochondrial function in pregnant women, and higher fasting blood sugar values in the infant [25].

Nonnutritive sweeteners can have effects on glucose homeostasis, weight gain, and body fat, especially if they are consumed over the long term [46]. The consumption of beverages with artificial sweeteners is associated with higher levels of glycated hemoglobin (HbA1c), triglycerides, and insulinemia, and a greater risk of obesity [25]. The consumption of aspartame by pregnant women may affect the structure, growth, and function of the placenta through stimulation of oxidative stress [47]. Berni Canani et al. (2024) have highlighted that the consumption of artificially sweetened drinks in pregnancy was related to a higher risk of asthma in offspring, and the consumption of baked and sugary foods was associated with a higher prevalence of food allergy at 1 year of age [48]. Gebremichael et al. (2024) examined the association between low-carbohydrate sweeteners consumption during pregnancy and child health outcomes in a review article, and concluded that the frequent consumption increased birth weight and the risk of overweight at different ages (6 months, 1 year, 3 and 7 years of age) [49].

#### 3.2.3. Impact of Protein Consumption During Pregnancy on Maternal and Offspring Health

Protein intake during gestation influences different processes in the offspring: fetal growth, metabolic programming, gene expression related to cell cycle regulation, muscle development, immune function, reproduction, and gut health, and it has an impact beyond birth, influencing postnatal growth trajectories and reproductive performance [50]. An adequate protein intake, higher in the last trimester of pregnancy, influences the duration of pregnancy, reducing the risk of preterm birth [35]. However, excessive protein intake may also have adverse effects, such as immune dysregulation and metabolic imbalances.

Low-protein diets lead to a lower mean islet size, a lower rate of beta-cell proliferation, and a higher incidence of beta-cell apoptosis in the islets of the offspring [43]. Maternal low-protein diets (under 12%) affect myostatin signaling and protein synthesis in the skeletal muscle of the offspring, with an impact on growth rate and muscle development. Costa et al. (2022) observed that maternal protein restriction during mid-gestation can limit muscle fiber formation in calves, an effect that persists throughout their lifetime and may lead to a slight increase in collagen accumulation in skeletal muscle, whereas protein supplementation during mid-gestation can promote muscle hypertrophy [55]. Maternal protein restriction can also lead to lower birth weights, altered metabolic programming, and compromised immune function in the offspring. The study by Jahan-Mihan et al. (2015), conducted in rats, demonstrated that adult rat offspring born to dams who received a low-protein diet during pregnancy had a lower body weight, metabolic abnormalities, and elevated blood pressure values [56]. The same authors in 2024, in a narrative review on the impact of maternal diet, mentioned that a low-protein diet can influence placental development and the transport of nutrients to the fetus, with IUGR as a consequence [52].

Amino acid levels influence fetal outcomes:

- The concentrations of some amino acids (serine, lysine, arginine, ornithine, proline) correlate positively with birth weight;
- Arginine is involved in fetal development and placental angiogenesis;
- Leucine supplementation can have effects on parameters related to obesity;
- Methionine restriction improves body weight gain, insulin sensitivity, and glucose metabolism;
- Tryptophan restriction modulates energy balance and induces weight loss;
- Glutamine mitigates inflammation and improves insulin sensitivity;
- Supplementation with glycine increases insulin levels, reduces systemic inflammation, and improves glucose tolerance;
- Supplementation with branched-chain amino acids (BCAAs), taurine, and cysteine during pregnancy prevents the onset of hypertension [51].

#### 3.2.4. Impact of Fast Food Consumption on Maternal and Offspring Health

The consumption of fast food, rich in fat, salt, and sugar, is associated with excessive weight gain in pregnancy, which raises the risk of GD, elevated blood pressure values, the risk of a non-elective cesarean section, and the risk of giving birth to a macrosomic fetus. Subsequently, a rapid increase in BMI in early childhood and in the risk of developing obesity could be observed [1,53]. The systematic review conducted by Ferreira et al. (2022) highlighted that the consumption of processed foods (sweets, pizza, hamburgers) can lead to excessive weight gain in pregnancy, as did the systematic review conducted by de Oliveira et al. (2022), which observed a positive correlation between the consumption of sugar, margarine, and snacks and an increased prevalence of excessive weight gain in pregnancy [53,57]. In addition, ultra-processed foods influence fetal and brain growth and development, increase the risk of LBW, preterm birth and of reduced verbal functioning in early childhood, promote oxidative stress and neuroinflammation, influence immune-related outcomes, immune cell development, and placental development and function, and have a negative effect on the intrauterine environment and on nutrient delivery to the fetus [58]. Increased salt consumption (over 5 g of sodium per day) may alter the function and metabolism of the placenta and may be associated with inflammation, hypoxia, and other pathogenic processes [59].

#### 3.2.5. Impact of Caffeine, Alcohol, and Tea Consumption on Maternal and Offspring Health

##### Caffeine

During pregnancy, caffeine is absorbed quickly and freely crosses the placenta, but its metabolism is considerably prolonged, owing to the significantly reduced enzymatic activity during pregnancy and to the inhibitory interaction between caffeine, estrogen, and progesterone, especially in the third trimester—returning to normal within a few weeks after birth. The half-life of caffeine in pregnant women is about 8.3 h, compared with 3.4 h in nonpregnant women, and in the third trimester it reaches 10.5–16 h [60]. Owing to these metabolic changes, caffeine accumulates in fetal tissues, increasing the risk of IUGR, miscarriage, or premature birth.

Numerous studies have examined the association between maternal caffeine consumption and disorders that can affect the health of infants and children, owing to the lipophilic properties of caffeine, which allow it to pass through the placental barrier into fetal blood [61]. One of the most commonly reported adverse effects related to caffeine consumption during pregnancy is the birth of an SGA newborn following exposure to excessive caffeine levels, the risk being dose-dependent. Caffeine intake exceeding 200–300 mg/day has been estimated to be associated with a 30–60% higher risk of SGA newborns, compared with those born to mothers whose caffeine intake throughout pregnancy remained below 50 mg/day. Caffeine intake over 151 mg/day (consumption of more than four servings of coffee per day) during the periconception period and in the first trimester of pregnancy was linked to recurrent miscarriages [60].

Given the established role of hypertensive disorders in pregnant women’s health and perinatal morbidity, several cohort studies have examined whether caffeine consumption during pregnancy influences the risk of gestational hypertension and preeclampsia, and it has been found that there is no significant correlation [39,62,63]. Likewise, Chen et al. (2022), in a meta-analysis that included 10 studies and 114,984 pregnant women, showed that there was no significant relation between caffeine consumption during pregnancy and the risks of preeclampsia or GD [64].

Anemia during pregnancy has been studied extensively, with the literature identifying the contribution of several factors that include caffeine consumption, maternal age, level of education, alcohol consumption, gestational age of the fetus, family size, and nutritional status. It was found that pregnant women who consumed caffeinated beverages (coffee or tea), even occasionally, had twice the risk of developing anemia during pregnancy compared with those who did not. The explanation given was that caffeine very possibly interferes with the absorption of non-heme iron in the gastrointestinal tract, which can decrease the bioavailability of maternal iron and contribute to the development of anemia during pregnancy [39].

An analysis of the Adolescent Brain Cognitive Development Study (ABCD Study), which included more than 9000 participants, showed less favorable outcomes in externalizing and internalizing behaviors, somatization, and neurodevelopmental behaviors in children prenatally exposed to caffeine, and a subsequent analysis of the ABCD dataset highlighted an association between caffeine consumption and behavioral externalizing difficulties, but not internalizing. Moreover, caffeine consumption during pregnancy increased the likelihood of more severe cases of oppositional defiant disorder, as well as cases with conduct problems, but the analysis concluded that caffeine exposure has no impact on cognitive difficulties in children [39,65]. The ABCD study on caffeine exposure during pregnancy signaled an association with an increased risk of higher BMI in childhood and sleep problems in childhood and adolescence [39,65,66]. Hu et al. (2024) suggested that drinking coffee during pregnancy, but not drinking tea, may increase the risk of developing brain tumors, especially glioma, in childhood [67].

##### Alcohol

The known and severe consequences of fetal exposure to alcohol through maternal consumption are grouped under fetal alcohol syndrome (FAS). The 2021 National Perinatal Survey reported that 3% of women consumed alcohol during pregnancy. It is important to note that, in general, alcohol consumption during pregnancy can often be associated with other substances, which also require systematic follow-up [68].

Alcohol freely crosses the placenta, altering neurogenesis through complex neurotoxic phenomena that are not yet fully elucidated. The maternal blood alcohol level will equal that of fetal blood, which is unable to metabolize it quickly owing to the immaturity of the liver enzymes. In addition, alcohol consumption causes placental toxicity, which leads to changes in oxygen transport, increasing the risk of early miscarriage, IUGR, or hypertension in pregnant women. Alcohol is teratogenic in the embryonic period and can determine possible organ malformations and neurotoxicity in the brain, the risk being independent of the type of alcohol consumed. The most severe manifestation caused by exposure to high doses of alcohol is FAS, which is associated with craniofacial dysmorphism, cardiac or renal malformations, growth retardation, microcephaly, and mental deficit [68].

##### Tea

Owing to the influence of traditional Chinese culture, the consumption of tea, especially green tea, is widespread compared with that of coffee. The combination of polyphenols and catechins in green tea provides neuroprotective properties. Arafa et al. (2024), in a meta-analysis, investigated the relationship between antenatal tea drinking and hypertensive disorders of pregnancy, observing that the highest consumption of antenatal tea was positively associated with this pathology, the mechanism being unclear [69].

**Figure 2:**
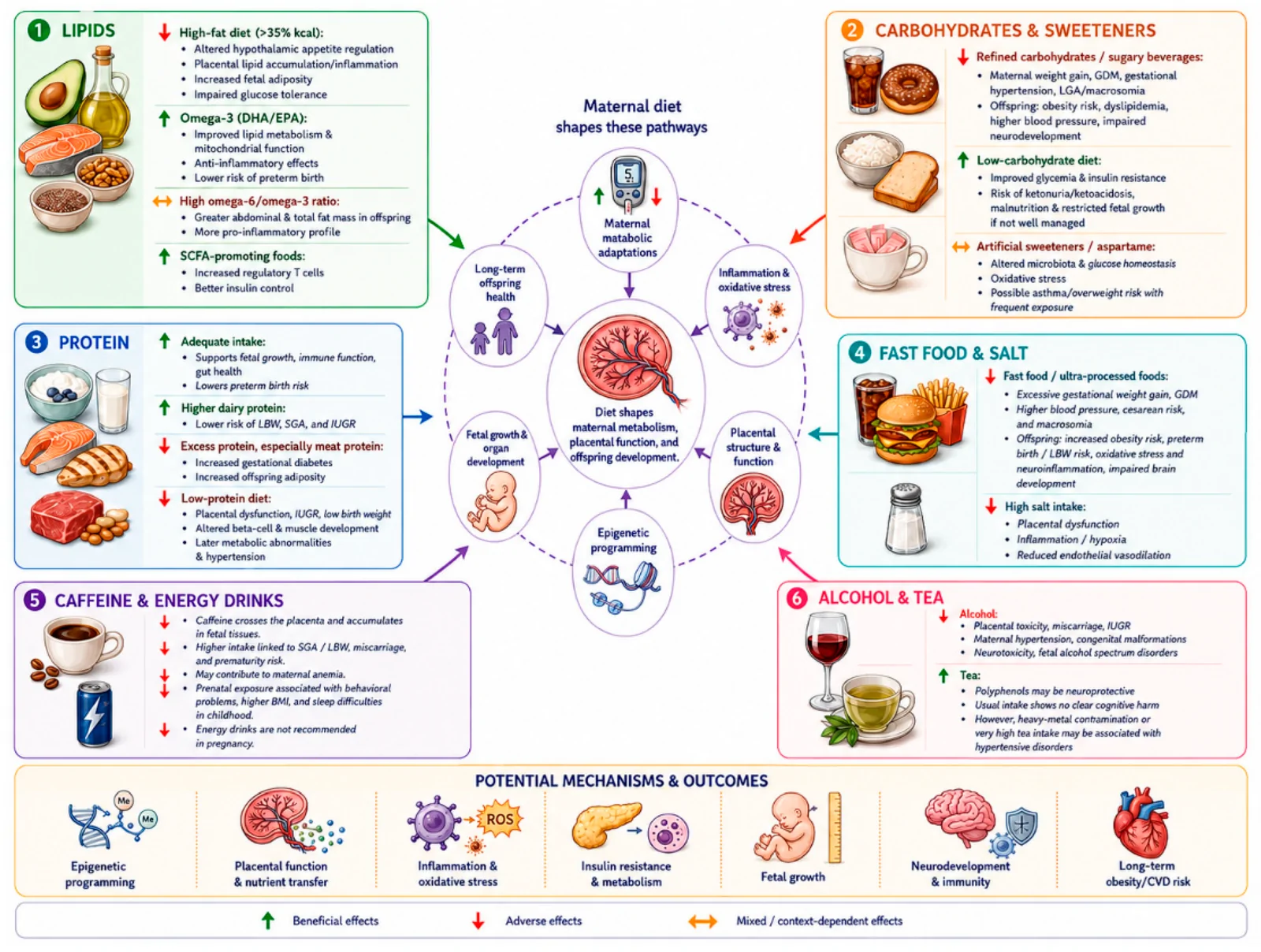
Impact of components of the diet on maternal and offspring health.

Figure 2 summarizes the impact of different components of the diet on maternal and offspring health.

Table 1 below presents the results of different studies on the influence of the consumption of certain nutrients on maternal and offspring health, by completing the data mentioned above.

### 3.3. Different Types of Diets and Their Influence on Maternal and Child Health

#### 3.3.1. The Mediterranean Diet

The nutrition of a woman of childbearing age can have benefits for her health, fertility, the course of pregnancy, and the offspring. A balanced, varied diet containing iron, calcium, zinc, vitamins D and B12, folic acid, and omega-3 can influence hormonal status, ovulatory function, and pregnancy, and can reduce the risk of chronic diseases. One example of such a diet is the Med diet, characterized by a high intake of antioxidants, fiber, and micronutrients and a low intake of carbohydrates and fat, which improves the metabolic profile, lowers IR and the risk of GD, and also has positive effects on the offspring, being associated with a lower risk of excess weight, a reduction in body fat percentage, a decrease in abdominal circumference, improved cognitive and executive function, and better communication abilities at 6 months of age [85,86]. The positive effects of the Med diet are due to the synergistic and interactive combination of nutrients and to the modulation of gene expression through epigenetic changes [87]. By modifying the expression of certain genes (NCK2, CLOCK) through various mechanisms (DNA methylation, histone modification, non-coding RNA), the Med diet is considered one of the most effective diets in disease prevention, reducing the risk of various comorbidities and obesity [1]. Furthermore, high compliance with this diet promotes a more favorable nutritional profile in pregnant women [88]. Zaragoza-Martí (2022) notes that the Med diet correlates negatively with the risk of obstetric pathologies and mortality, the prevalence of coronary disease and other cardiovascular diseases, thrombotic risk, the incidence of certain types of cancer (breast, colorectal), type 2 diabetes, and hip fractures, owing to its high content of fiber, antioxidants, and low-glycemic-index foods and its high content of MUFAs and PUFAs [89]. The diet during pregnancy also influences the development of the offspring’s immune system and modulates the risk of developing atopic dermatitis, the mechanisms involving the GM and immunomodulation via vertically transferred metabolites [16]. The Med diet enhanced mucosal immunity, as demonstrated by an increase in fecal IgA and in the level of Ig-coated bacteria [15].

Owing to its high content of omega-3 (EPA, DHA) as well as MUFAs, vitamins, carotenoids, and polyphenols, it has anti-allergic and anti-inflammatory effects, providing protection against allergies [87]. The studies by Méndez et al. (2021) and Schneider et al. (2024) report that the polyphenols in fruit and vegetables amplify the anti-inflammatory effects of the omega-3 fatty acids in fish, facilitating the absorption of fat-soluble vitamins—which is essential for immune tolerance and the reduction in atopy risk—an absorption that is also increased by MUFAs and PUFAs [90,91]. The consumption of fruit (oranges, cherries, grapes) and tomatoes provides an intake of antioxidants (glutathione, vitamins C, E, and A, carotenoids, and polyphenols), suggesting a protective effect against asthma, atopy, and allergic rhinitis. Flavonoids and polyphenolic plant metabolites from fruit, vegetables, nuts, and seeds have antioxidant, anti-inflammatory, and anti-allergic effects, contributing to a protective bronchial effect and a reduced risk of asthma and atopic dermatitis. Soy isoflavones contribute to reducing the number of severe asthma exacerbations [92]. Supplementation with malic acid, which is found in unripe fruit, increases antioxidant capacity and modifies the characteristics of muscle fibers through a slow-twitch to fast-twitch transition in the skeletal muscles of the offspring [93].

The Med diet has benefits for placental development and improves placental flow. Polyphenols improve the transport of various bioactive compounds to the placenta. Epigallocatechin gallate—a polyphenol contained in tea leaves and coffee—has a favorable effect in the alleviation of oxidative stress in trophoblast cell lines. Stilbenes, particularly resveratrol, exhibit antioxidant and anti-inflammatory effects in the placenta, safeguarding human trophoblasts against H_2_O_2_-induced oxidative stress. Dietary fiber supplementation during pregnancy modulates the gut microbiota, improves insulin sensitivity, and increases placental vascular density. Choline (vitamin B4) has beneficial effects on placental function, modulating angiogenesis, inflammation, and macronutrient transport, and, by modulating the expression of genes involved in the hypothalamic-pituitary-brain axis, can influence the fetus’s stress response [47]. The Med diet during pregnancy correlates positively with placental TL, while a diet rich in carbohydrates, fat, and alcohol, as well as maternal infections, correlates negatively with TL. The Med diet reduces the risk of GD, preterm birth, and urinary tract infections, increasing TL. It is associated with better fetal growth, a better course of pregnancy, a lower incidence of certain complications, and better long-term health [35,36]. Low TL is related to an increased risk of cardiovascular, pulmonary, and neurodegenerative diseases. The intake of vitamin C, with its antioxidant role, during pregnancy correlates with greater neonatal TL [35].

The Med diet, rich in MUFAs and omega-3 PUFAs derived from olive oil, oily fish, nuts, and seeds, reduces inflammation and supports brain development in the offspring, especially through its DHA content [86]. During pregnancy, an increased intake of omega-3 LCPUFAs through supplementation with 0.1 g of EPA and 0.8 g of DHA daily can contribute to lowering the risk of sensitization to hen’s egg and of developing IgE-mediated eczema, also having positive immune effects, the mechanisms of which are not well understood. Omega-3 supplementation in the pregnant woman can influence the Th1/Th2 ratio in the infant by modulating the DNA methylation levels of the genes encoding these cytokines [45].

Extra-virgin olive oil (EVOO)—the main source of fat in the Med diet, of which 80% is MUFAs (especially oleic acid)—contains a high percentage of antioxidants (phenolic acids, lignans, flavonoids) with an impact on the mother’s immune system, the gene expression of IgA in the mammary glands of rats being boosted by the consumption of this type of oil, as shown by Zhan-Dai et al. (2024) in their study conducted on rats [94]. Its consumption has effects on the immune system, with the IgG2c concentration in plasma increasing, which might contribute to enhanced systemic and intestinal immune regulation in the offspring [95]. An increased intake of oleic acid—the main MUFA in olive oil—correlates negatively with the onset of asthma [92]. The consumption of olive oil increases the activity of enzymes involved in antioxidant activity (glutathione peroxidase, catalase, superoxide dismutase), with polyphenols of the oleuropein type being able to act as regulators of redox-sensitive placental physiology, thereby influencing trophoblast functional behavior. It also has anti-inflammatory effects through the reduction in inflammatory biomarkers, as well as antimicrobial and GM-modulating effects [36,94]. Guasch-Ferré et al. (2017) noted that polyphenols can reduce IR and glycemic load, stimulate insulin secretion, modulate glucose release, and contribute to the control of excessive weight gain, while Weickert et al. (2008) noted that an increased intake of fiber reduces inflammation and favorably influences glycemic control [96,97,98]. The consumption of more than 5 g/day of olive oil plays a role in the prevention of SGA and GD [99]. Supplementation of the pregnant woman’s diet with EVOO reduces adiponectin in the offspring, with the levels of leptin and the leptin: adiponectin ratio remaining unchanged, and it can increase the phenolic content of human milk [95,100].

The favorable effects of this diet on the offspring include a reduction in blood pressure, leptin, and adiposity, a lower risk of an accelerated growth pattern and of higher birth weight, and a lower risk of developing obesity at 4 years of age. The Med diet has an anti-obesogenic effect on brown adipose tissue by upregulating the expression of certain genes (Cidea, Ucp-1, Gpr43, Prdm16). With regard to the effects of the pregnant woman’s Med diet on epigenetic changes in the offspring, there are not many studies [1]. Antioxidants contribute to improving metabolism in the offspring through the improvement of the values of leptin, adiponectin, triglycerides, and IR [4].

Over the past 50 years, the pattern of the Med diet has changed, with its adaptation toward patterns specific to the Western diet (WD). The reduction in the intake of omega-3 fatty acids may contribute to an increase in the prevalence of diseases characterized by wheezing, and an increased omega-6:omega-3 ratio in the maternal diet, as well as the presence of maternal asthma, raises the risk of wheezing or asthma in the offspring. The protective effects of the Med diet can be reduced by exposure to environmental contaminants present in various foods (fruit, vegetables, cereals, seafood, fish). Therefore, supplementation with omega-3 in the third trimester of pregnancy reduces the risk of lower respiratory tract infections and of persistent wheezing [92].

While findings on the Med diet and GM are promising, most evidence comes from observational cohort studies, which are susceptible to confounding by socioeconomic status, educational level, healthcare access, physical activity, and other healthy lifestyle behaviors that often cluster with Med diet adherence. The definition of “Mediterranean diet” varies by population and may include different proportions of olive oil, fish, and whole grains, affecting reproducibility. Adherence to the Med diet is assessed using different tools across studies, including food frequency questionnaires, Med Diet Scores, and dietary indices, which limits comparability. Fourth, few randomized controlled trials have specifically tested Med diet interventions during pregnancy with microbiome or long-term offspring outcomes as primary endpoints. Therefore, while the Med diet is associated with favorable GM patterns, causal inference and generalizability require further interventional studies.

#### 3.3.2. The Western Diet

The WD is a dietary pattern characterized by high intakes of pre-packaged foods, refined grains, red meat, processed meat, high-sugar drinks, candy, sweets, fried foods, conventionally raised animal products, high-fat dairy products, and high-fructose products [101]. This high-fat diet induces alterations of placental structure and metabolism, promotes the development of dysbiosis and obesity in the pregnant woman, and promotes disturbances in neurological development in the offspring (autism spectrum disorder—ASD, attention deficit hyperactivity disorder—ADHD, depression, anxiety, schizophrenia). Intestinal dysbiosis associated with inflammation can affect neurological development, and the social deficits observed in the offspring of mothers who consumed large amounts of fat appear to be mediated by the gut microbiota–brain axis. Studies conducted on the offspring of mice concluded that a maternal diet rich in saturated fatty acids can contribute to a decreased hippocampal size, and that a diet poor in PUFAs can alter the function and morphology of the neurons in the hippocampus. Studies on the role of a high-fat maternal diet in the emergence of anxiety, especially in the period of higher neuronal plasticity, have yielded variable results. Kang et al. (2014) and Glendining et al. (2018) observed the presence of anxiety in juvenile and adult females, while more recent studies (Lippert et al., 2020; Bordeleau et al., 2021), showed no differences [102,103,104,105]. A WD rich in omega-6 and poor in omega-3 can affect neocortical neurogenesis, with long-term effects on the offspring’s brain, also increasing the risk of anxiety and depression and of reduced sociability, and promoting the development of food allergies [28,45]. The WD can affect the offspring’s neuronal connections, with negative and permanent effects on certain metabolic processes [106]. Owing to its high content of fat and also carbohydrates and its low content of fermentable fiber, it can increase the risk of type 2 diabetes, obesity, and other metabolic diseases and lower the level of SCFAs [3,7].

The WD contains foods with pro-inflammatory effects (red meat and simple carbohydrates). The calculation of the dietary inflammatory index (DII) of this diet during pregnancy and early childhood revealed that elevated DII values are associated with higher values of BMI, abdominal circumference, and skinfolds. Luszczki et al. (2024) confirmed that the WD can increase weight and the risk of obesity in the offspring in infancy and up to 10 years of age [1]. The diet’s high content of saturated fatty acids and of calorie-dense, ultra-processed foods containing refined carbohydrates leads to an increase in reactive oxygen species (ROS), unlike a diet rich in antioxidants (unsaturated fatty acids, micronutrients), which maintains oxidative balance [36]. A high content of ultra-processed foods and a low content of nutrient-dense foods are associated with increased inflammation, a risk of unfavorable pregnancy outcomes, cardiovascular risk, skeletal impairment, and an inadequate status of vitamins and minerals [86]. Processed foods (sugar, refined grains) correlate with shorter TL and may be associated with LGA births [2,19]. Increased sugar consumption during pregnancy is associated with higher BMI values and a higher rate of weight gain in children between 2 and 4 years of age. Unlike the consumption of low-glycemic-index foods, which is correlated with lower weight, height, and arterial wall thickness, the consumption of high-glycemic-index foods in the first trimester of pregnancy is linked with an increase in total, visceral, and abdominal fat in the offspring [1].

Compared with diets rich in fresh, unprocessed foods, the WD—rich in processed and packaged foods—increases exposure to phthalates, chemical compounds used in the production of plastic and other commercial products. This exposure increases the risk of impaired reproductive health, preterm birth, and low birth weight [107].

#### 3.3.3. The Vegetarian Diet

A diet rich in plant proteins, PUFAs, and fiber lowers the incidence and severity of infections in offspring during the first year of life, whereas a diet rich in animal protein, MUFAs, and cholesterol leads to a reduction in the level of IgA in human milk, increasing the risk of infections. LCPUFAs are transferred through the placenta and influence the development of the fetus [15]. Flavonoids from vegetables and fruit reduce oxidative stress. Carotenoids (lutein, beta-carotene) play a role in vascular and oxidative balance, contributing to better blood vessel function in the last trimester of pregnancy as well as after birth [36].

Meulenbroeks et al. (2024), in a systematic review of six articles that included vegan and omnivore participants, observed that the children whose mothers were vegan were SGA, probably owing to the lower protein intake, presenting a risk of developing cardiovascular disease in adulthood, and that only a small percentage of vegan pregnant women presented excessive gestational weight gain with an increased risk of GD, LGA, macrosomia, hypertension, and cesarean section [108]. In another systematic review, Papadopoulou et al. (2025) observed that mothers who followed a strict vegetarian diet (Veg diet) had a higher risk of having SGA children, and that vegan mothers had a lower risk of excessive weight gain during pregnancy, with no significant differences in the risk of developing GD [109]. In contrast, Palma et al. (2023), in a literature review, noted that the consumption of a vegan diet during pregnancy is associated with a lower risk of GD, but—as in the previous article—with a lower risk of excessive weight gain, as well as of hypertension [110]. The results regarding the child’s birth weight were contradictory, it being considered that this may also be influenced by other factors (genetic, socioeconomic), not only by the maternal diet [110]. Racisz et al. (2025), in a review of the scientific literature, showed that pregnant vegetarian women had a lower or the same BMI as omnivorous women, a lower risk of type 2 diabetes mellitus and cardiovascular disease, and a high risk of nutritional deficiencies, especially of vitamin B12, iron, and DHA [111]. The Veg diet, as Jahan-Mihan et al. (2024) noted in their narrative review, supports fetal development and reduces the risk of chronic diseases; however, owing to the lack of certain nutrients (vitamin B12, omega-3, iron, zinc, and iodine), it can have unfavorable effects on immune function, neurodevelopment, and growth [52].

#### 3.3.4. The Ketogenic Diet

A very low-calorie ketogenic diet (KD) cannot currently be recommended in pregnancy, as the restrictions can negatively affect both the mother and the offspring [13]. During pregnancy, ketone bodies reach the same concentrations in the circulation of the mother and the fetus by crossing the placenta.

Studies on animal models have shown that the KD:

- Alters fetal growth trajectories;
- During pregnancy, decreases or increases embryo size depending on the stage of development, and can affect brain development and cause epigenetic changes in obesity gene expression;
- Increases maternal and fetal plasma leptin levels, without an effect on insulin levels;
- Has no effect on the length of pregnancy;
- Leads to a drop in glucose levels and an increase in ketones and triglycerides, without ketoacidosis;
- Decreases brain weight and impairs brain development;
- Causes placental failure and fetal growth restriction;
- In female fetuses, leads to a lower weight and a higher ear morphological grade;
- Leads to a relatively larger heart and a smaller brain, hypothalamus, and cervical spinal cord;
- Delays the development of neurological, reflex, and somatic responses;
- Reduces neuronal density in the prefrontal cortex and dentate gyrus;
- Can improve sociability and reduce depression- and anxiety-like behaviors;
- Has a protective effect on symptoms of autism spectrum disorders in the offspring;
- Reduces lifespan and leads to a late-onset increase in body mass [112,113].
- In human studies, which are limited, the KD:
- May be associated with cardiac arrhythmias, hypoxia, or fetal death;
- Increases maternal metabolism and insulin requirements, with an increased risk of euglycemic ketoacidosis as a consequence;
- Increases genomic DNA methylation;
- Leads to good control of epileptic seizures in pregnant women with epilepsy and alterations in brain structure, in neurological development, and in later behavioral changes;
- Is associated with chronic inflammation in the fetal environment;
- Promotes overgrowth and increases adiposity, with a subsequent risk of obesity [25,58,113].

**Figure 3:**
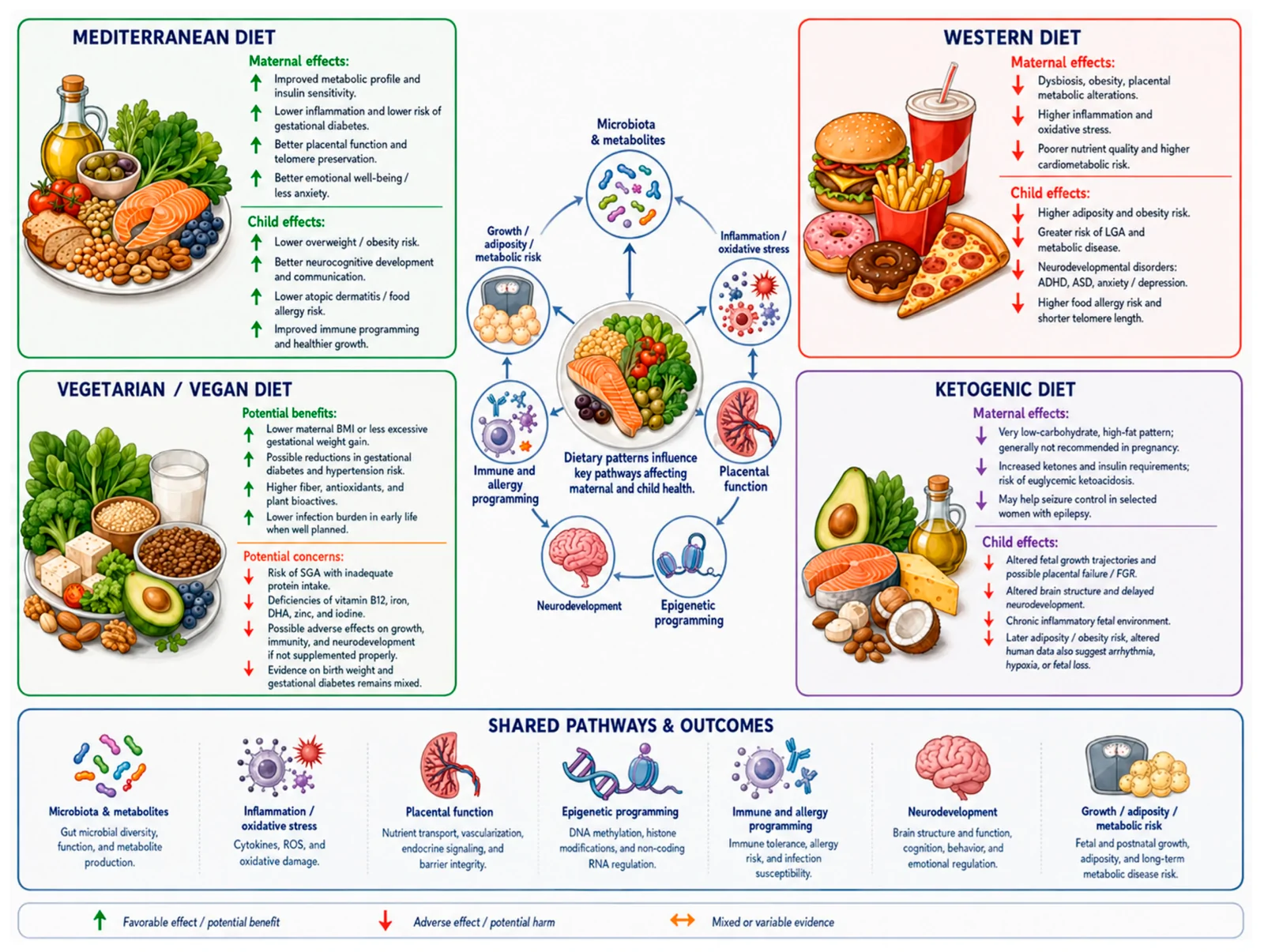
Different types of diets and their influence on maternal and child health.

Figure 3 schematically shows the influence of different types of diets on maternal and child health.

Table 2 presents the results of some studies on the influence of the consumption of different diets on maternal and offspring health.

Among the dietary patterns reviewed, the Mediterranean diet has been the most extensively studied in relation to maternal and offspring microbiota, although direct causal evidence during pregnancy remains limited. The WD is most consistently associated with adverse outcomes; the Veg diet is safe if it is carefully planned, while the KD cannot currently be recommended in pregnancy due to insufficient human safety data; most available evidence is derived from animal studies and its use should be approached with caution. Heterogeneity in study design limits direct comparison between diets. Studies about the KD are restricted to animal models and there are no studies directly comparing these four diets.

## 4. Discussion

Pregnancy is a period in which nutritional requirements increase in order to support the development of the maternal organs and the fetus. The results of different studies are heterogeneous, owing to the different study designs, the influence of environmental confounders, the method of quantifying intake, and differing dietary habits [124]. Given the narrative nature of this review, the strength of evidence varies across dietary factors. Human randomized controlled studies and prospective cohorts provide the strongest data for outcomes such as gestational weight gain and SGA. Mechanistic studies in humans support biological plausibility for pathways such as SCFAs production and inflammation, while studies on animals should be considered hypothesis-generating and require validation in human trials before clinical recommendations can be made.

The diet–microbiota interaction in pregnancy is a rapidly expanding field, with major implications for maternal–fetal health. The data presented in this review confirm that the maternal microbiota is remodeled by the hormonal, metabolic, and immune changes in the pregnancy period, and that the pregnant woman’s diet is an important modulating factor in this remodeling [5,6].

The data in the current literature regarding GM composition over the course of pregnancy are variable: some studies report trimester-by-trimester changes, such as an increase in *Pseudomonadota* and *Actinomycetota* in the first trimester and a reduction in alpha-diversity in the third trimester, while other studies support a relative stability of the GM [7]. One thing is clear, however, regarding the role of vertical transmission: the placental intrauterine microbiota, the microbiota of human milk, and early neonatal colonization are all influenced by the maternal diet [8,11]. Diet-induced maternal dysbiosis may constitute the molecular basis for the transgenerational transmission of obesity and other metabolic diseases [1]. Beyond the outcomes discussed above, the GM has also been implicated in other chronic conditions (inflammatory bowel disease, autoimmune disorders, rheumatoid arthritis, and certain cancers). These were not the primary focus of this review due to the paucity of pregnancy-specific human data and because antenatal dietary exposures are less well established as direct risk factors compared to postnatal exposures. Additionally, conditions such as depression and anxiety, while linked to the gut–brain axis, have heterogeneous etiologies that extend beyond the perinatal period.

Diets rich in fiber, polyphenols, and fermented foods increase the abundance of SCFAs-producing species and reduce species with pro-inflammatory potential, having a protective role against GD and preeclampsia through the reduction in IR, the lowering of placental inflammation, and the increase in IL-10 [12,23,125]. The Med diet and the Veg diet, which are also rich in fruits, vegetables, fibers, and bioactive compounds, are associated with increased beta diversity and a favorable microbial profile in meconium; however, some studies have found no changes in maternal alpha diversity [10,14]. Studies support the protective beneficial effect of polyphenols on transgenerational health: they reduce the risk of GD and improve antioxidant status and the lipid profile [126,127]. However, excessive doses can have adverse effects through epigenetic changes in the fetal developmental trajectory [127]. Studies on quercetin and resveratrol support their protective potential against obesity, hypertension, and IR induced by a maternal high-fat diet, through DNA methylation at the level of the leptin gene [128]. Nevertheless, there is a need to define the “therapeutic dose” of polyphenols during pregnancy, given their low bioavailability and their interactions with the microbiota.

A maternal diet rich in fat, especially in saturated fatty acids, induces dysbiosis that can persist until 6 weeks of age and is associated with a reduction in the level of *Bifidobacteria* in human milk [8,12,28]. This maternal high-fat diet can alter the child’s behavioral phenotype and can affect the HPA axis, with consequences in adolescence (depression) [3]. It has also been observed that the type of fat consumed has different effects: for example, MUFAs and PUFAs, especially omega-3, increase the levels of *Ruminococcaceae*, *Lachnospira*, and *Bifidobacteria*, with anti-inflammatory effects at the placental level [22,36]. Elevated omega-3 concentrations are associated with a lower body fat percentage in the child, while an increased omega-6/omega-3 ratio promotes abdominal adiposity [1]. The proposed mechanisms include the modulation of lipid metabolism, mitochondrial function, and the reduction in oxidative stress. Among the fat-soluble vitamins, an increased intake of vitamin D has been paradoxically associated with a decrease in alpha diversity and an increase in *Proteobacteria*, suggesting that the dose–response relationship for fat-soluble micronutrients requires clarification [22]. An adequate protein intake, especially in the last trimester, correlates with a prolongation of the duration of pregnancy and a reduction in the risk of preterm birth, while a high-protein, low-carbohydrate diet decreases *Roseburia*, and protein deficiency reduces diversity and lowers the genera involved in SCFA production [26,30,35].

With regard to carbohydrates, natural sugar has the opposite effect to sweeteners, increasing *Bifidobacterium*, while the latter are associated with a reduction in *Bacteroides* in the infant and with an increase in BMI at 1 year [13]. An excessive intake of refined carbohydrates increases the risk of LGA, GD, and obesity in both mother and child [25]. Furthermore, a diet rich in refined cereals and sugar is associated with a shortening of telomeres in cord blood, a marker of cellular aging [35]. Severe carbohydrate restriction, although it improves IR and glycemia, can reduce fertility, induce ketonuria, and affect retinal microcirculation and mitochondrial function [25]. Animal studies have shown that aspartame increases oxidative stress, maternal weight, and the risk of asthma in offspring through a Th1/Th2 imbalance [72]. In humans, the frequent consumption of sweetened beverages, with or without sugar, correlates with elevated HbA1c, triglycerides, and a risk of macrosomia [80].

Fast food and foods high in salt represent a combination with pro-inflammatory effects, cumulative metabolic risk, and transgenerational effects. The results of different studies support the association with excess weight in pregnancy, GD, hypertension, and the need for cesarean delivery, and in the child, a rapid increase in BMI in early childhood and a risk of developing obesity [1,53].

Caffeine crosses the placenta, and its half-life increases from 3.4 h to 10.5–16 h in the third trimester of pregnancy. An intake of over 200–300 mg/day increases the risk of SGA by 30–60%, and a consumption of over 151 mg/day in the periconception period is associated with a risk of recurrent miscarriage. Relatively recent studies have confirmed an increased risk of SGA even with an intake of 51–200 mg/day [60]. In the long term, it has been observed that prenatal exposure to caffeine correlates with elevated BMI and sleep and behavioral disturbances in the child [65,66]. According to the study by Struniewicz et al. (2025), caffeine does not significantly affect alpha diversity or species abundance, although studies on animal models report changes in the levels of *Enterobacteriaceae* and *Clostridium* [39,41]. Owing to their high caffeine content, energy drinks are contraindicated in pregnancy [60].

Alcohol, unlike caffeine, has clear negative effects, so it remains subject to a “zero” recommendation, there being no safe level of consumption, and its teratogenic effects are independent of the type of alcohol [13,31]. The preconception period is the ideal time to prevent the effects of maternal and paternal alcohol consumption on the fetus, but unfortunately many pregnancies are often unplanned [68]. In the case of tea consumption, the heavy metal content of *Camellia sinensis* and the association of consumption in large quantities with hypertension call for caution on the part of pregnant women [69,77].

The analysis of the literature confirms that the overall maternal dietary pattern is more important than the isolated intake of nutrients in determining the course of pregnancy and the long-term health of the offspring [1,87]. The Med diet can be considered a complex nutritional whole-diet model that contains multiple bioactive components with influences on metabolic and redox-sensitive pathways [36]. The Med diet represents the “anti-inflammatory” diet model, with high adherence being associated with a reduction in IR, a lower risk of GD, obstetric pathologies, and mortality, as well as a lower risk of obesity and better cognitive and executive function in the child [48,85,88,89]. While EVOO, the main source of MUFAs, increases the activity of antioxidant enzymes, modulates the GM, and increases the phenolic content of human milk, the MEDALION study shows that not all components of the Med diet have a protective effect: the high consumption of fish, red meat, and poultry is associated with an increased risk of allergies in the offspring, while fruit and full-fat dairy have protective effects. Likewise, the “westernization” of the Med diet, through the reduction in omega-3 and the increase in the omega-6/omega-3 ratio, diminishes the protective effects against wheezing and asthma [94,100,115]. According to the study by Luszczki et al. (2024), the specific epigenetic changes induced by the Med diet in humans are still limited [1]. Specific to dietary patterns, the apparent benefits of the Med diet must be interpreted cautiously. The majority of supporting data are observational and may reflect broader socioeconomic and lifestyle factors rather than a direct effect of diet alone. Heterogeneity in adherence assessment and limited randomized controlled trials data in pregnancy further constrain conclusions. If the Med diet has anti-inflammatory properties, the WD is the opposite, with its pro-inflammatory pattern having a neuro–metabolic impact [101]. The consumption of a Western-type diet is associated with structural and metabolic placental alterations, maternal dysbiosis, obesity, and an increased risk of GD and cardiovascular disease in the mother, and with impairment of the child’s neurodevelopment [3,36]. Furthermore, maternal consumption of a WD has been associated with an adverse inflammatory and metabolic phenotype in offspring in animal studies, suggesting potential programming effects that require confirmation in human cohorts. The Veg diet, rich in plant proteins, PUFAs, and fiber, has metabolic benefits and is correlated with a reduction in the risk of infections in the infant during the first year of life and with a lower risk of GD and maternal excess weight [15,110]. However, relatively recent studies on the vegan diet raise an alarm, since it is associated with an increased risk of SGA in the offspring, probably owing to insufficient protein and caloric intake, as well as with nutritional deficiencies, especially of vitamin B12, iron, and DHA, making permanent monitoring necessary [108,109,110,111]. The KD is contraindicated in pregnancy, as severe carbohydrate restriction can induce ketonemia, oxidative stress, and a glucose deficit in the fetal brain, with long-term neurological consequences [13].

**Figure 4:**
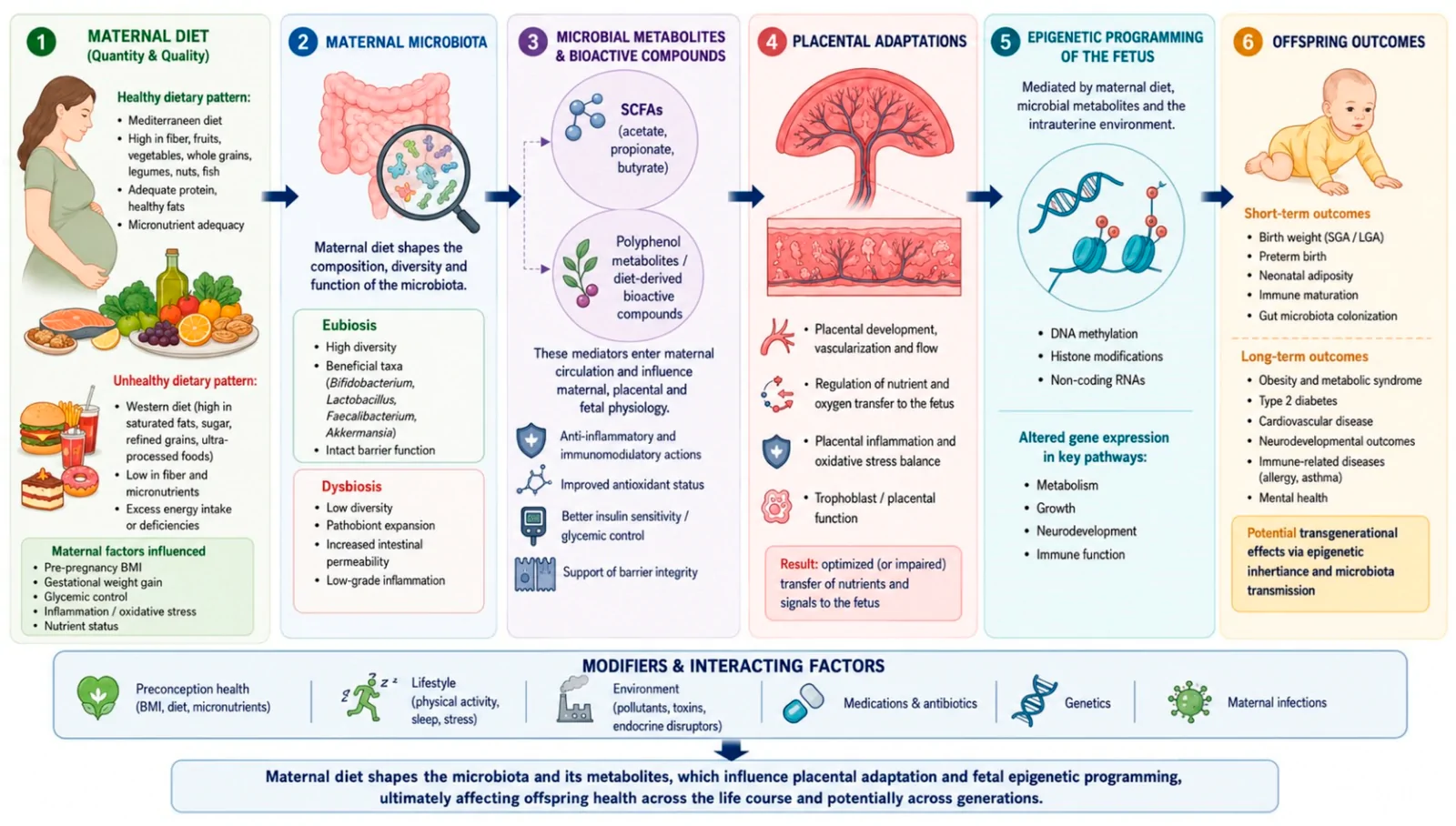
Proposed integrative pathway linking maternal diet to offspring health.

To integrate the mechanisms discussed above, we propose a conceptual model linking maternal dietary intake to offspring health outcomes—Figure 4.

Regarding the actual dietary interventions and their effect on clinically meaningful outcomes, the most consistent intervention data come from trials on the Mediterranean dietary pattern. The findings suggest potential clinical utility, but most trials were conducted in Mediterranean populations, limiting generalizability. There is an absence of trials evaluating other dietary patterns (KD, high-protein, intermittent fasting diets) in pregnancy. The available data for these patterns are almost entirely preclinical and their effects on clinical outcomes such as GDM, hypertensive disorders of pregnancy, preterm birth, birth weight, and long-term offspring metabolic health are unknown. Overall, the current literature is limited by small sample sizes, short follow-up, heterogeneity in dietary definitions, these being a barrier to translating biological insights into evidence-based dietary guidelines for pregnancy.

We acknowledge that the current literature is largely descriptive. The available evidence consists primarily of observational human studies and preclinical animal models, with few randomized trials. While we have summarized findings from systematic reviews, observational studies, and mechanistic investigations together, we now emphasize in Table 1 and Table 2 and throughout the text the considerable differences in their evidential strength and the limitations inherent to each design.

### Limitations, Gaps, and Future Directions

Unlike previous reviews that have addressed maternal diet or microbiota in isolation, this review integrates evidence across dietary patterns, macronutrients, and the microbiota within a DOHaD framework. A key contribution is the explicit stratification of evidence by study type, which reveals that while associations between diet and maternal outcomes are supported by human trials, most data linking diet to long-term offspring programming remain preclinical.

The limitations of this review may be represented by the inclusion of articles predominantly published in the last 5 years, available in full text on PubMed and Google Scholar, without the consultation of other databases as well.

This literature review highlights several gaps:

- Most of the studies were observational studies, with randomized studies on diet in pregnancy being rarer. There is a lack of studies stratified by maternal phenotype, metabolic status, BMI, and ethnicity, which could explain the variability of the microbial response to the same diet. The response to the same diet differs in normal-weight pregnant women compared with obese pregnant women or women with GD;
- Most of the data come from the last two trimesters of pregnancy, although metabolic programming begins before conception, and microbial colonization begins in utero and is probably also influenced by the preconception diet;
- Reports of transgenerational effects are derived from animal models or from observational studies with short to medium-term offspring follow-up. These gaps should guide future research and antenatal guidelines that should include: randomized and mechanistic studies in human models, the identification of predictive microbial biomarkers for pregnancy complications, long-term follow-up of offspring to clarify causal relationships and the development of personalized dietary recommendations according to the maternal microbial profile. Nutritional counseling for pregnant women, such as that provided in maternity wards, should focus on raising awareness of the safety limits for certain nutrients and on motivating the pregnant woman to follow them, especially targeting women at high risk of excessive consumption.

## 5. Conclusions

The present review confirms that maternal nutrition is not merely energy support for pregnancy, but a factor in fetal programming with a long-term impact on the child, in line with the DOHaD concept. The periconceptional period and the first trimester represent the “critical window” for intervention; however, most studies focus on the second and third trimesters. The maternal diet may act as an epigenetic and metabolic influence and has been proposed to have potential transgenerational effects, primarily based on findings from animal models. The quality of the diet matters more than the quantity. There are no universally “forbidden foods”, but the type of diet influences risk, with a nutrient-dense, anti-inflammatory, and sustainable diet throughout the periconceptional period and pregnancy having positive influences. Correct maternal nutrition does not mean “eating for two”, but “eating intelligently for two generations”. Optimizing the diet in the periconceptional period and during pregnancy represents an accessible and effective form of preventive intervention, but it requires personalized recommendations and structured nutritional education begun as early as the preconception period.

## Figures and Tables

**Table 1:** Influence of consumption of some nutrients on maternal and offspring health.

Dietary Pattern/Authors	Study Design & Model	Evidence Level	Key Findings	Key Conclusion/Clinical Relevance
**Animal studies**				
**High-fat diet** **Ceasrine et al., 2022** [44]	Animal study C57BL/6J mice, n = 6 placentas/group	Low Preclinical	Maternal high-fat intake ↑ placental lipid accumulation → TLR4 activation → innate immune response. ↑ Triglycerides in placenta → systemic inflammation in newborns. Disrupted fetal brain 5-HT in males.	Provides mechanistic plausibility for inflammation and neurodevelopmental effects. These findings are based on a small sample of C57BL/6J mice and require validation in human studies before clinical implications can be drawn.
**High-protein diet** **Freire et al., 2024** [70]	Animal study Rats, 30 dams	Low Preclinical	In utero exposure to high-protein diet ↑ offspring fat mass and body weight in early life. Combined with Western diet → long-term effects on body composition and metabolism.	Suggests maternal protein excess may program offspring obesity risk. Limited by small sample, undefined diet composition, and lack of human data. Warrant confirmation in clinical studies.
**High-fiber + polyphenol diet** **Ceballos-Sánchez et al., 2025** [71]	Animal study Wistar rats, 40 females	Low Preclinical	↑ Expression of genes related to immune response, antioxidant status, lipid metabolism and growth.	Indicates potential protective role of fiber/polyphenols on placental gene pathways. Limited by animal model and absence of phenotypic/offspring outcome data. The clinical relevance during human pregnancy remains to be determined.
**Artificial sweetener: Aspartame** **Chuang et al., 2025** [72]	Animal study BALB/c mice Control: 5 dams, 37 pups Aspartame: 3 dams, 19 pups	Low Preclinical	Maternal Aspartame exposure during pregnancy ↑ body weight in dams and offspring. ↑ TH2 and ↓ TH1 cytokines in offspring. No significant change in offspring GM composition or alpha-diversity.	Suggests Aspartame may skew fetal immune programming toward Th2 and contribute to weight gain. Limited by very small sample size, use of Th2-biased mouse strain, and lack of dose translation. Generalizability to humans is unclear.
**Human studies**				
**Protein intake & birth weight** **Yang et al., 2022** [73]	Prospective Cohort n = 7310 pregnant women	Moderate Observational	Higher intake of dietary protein associated with ↑ birth weight and ↓ risk of LBW, SGA, IUGR.	Suggests protein may be protective for fetal growth. Limited by self-reported diet and residual confounding. RCTs needed before clinical recommendations.
**Protein patterns & GDM risk** **Wu et al., 2022** [74]	Prospective Cohort n = 1014 pregnant women	Moderate Observational	Higher dietary intake of protein during mid-pre was associated with an increased risk of GD	Indicates timing and amount of protein may matter for glucose metabolism. It is limited by potential confounding and lack of data on protein source, and cannot establish causality.
**High coffee intake & LBW** **Mannucci et al., 2022** [75]	Prospective Cohort n = 5405 pregnant women	Moderate Observational	A significant association between drinking three or more coffees per day (about 366 mg of caffeine per day) and LBW in babies of gestational age over 37 weeks (*p* = 0.018)	Supports current guidance to limit caffeine in late pregnancy. Limited by self-reported intake and potential confounding. Causality not established.
**Prenatal caffeine & offspring metabolism** **Agarwal et al., 2022** [76]	Prospective Cohort n = 11,875 children, age 9–11 years, with recalled prenatal exposure	Low Observational	Excessive prenatal caffeine exposure might be detrimental to frontal lobe development and altered reward sensitivity to food, increasing risk for elevated total sugar intake and obesity. Recommendations to limit caffeine intake during pregnancy.	Raises concern for long-term neurobehavioral and metabolic programming. As an observational study relying on recalled prenatal exposure and with substantial postnatal confounding, it cannot establish causality and requires replication
**Heavy metals from tea** **Shah et al., 2022** [77]	Cross-sectional n = 400 pregnant women	Low Observational	Positive correlation between aluminum, lead, mercury and zinc and fetal weight. Negative correlation between copper, lead, aluminum, cadmium, mercury, zinc and foot length Negative correlation between aluminum and copper and chest circumference	Suggests tea-related heavy metal exposure may influence fetal growth parameters. Limited by observational design, self-reported tea intake without biomarkers, multiple testing, and potential confounding. Causal interpretation not possible.
**Preconception caffeine & birth defects** **Williford et al., 2023** [78]	Prospective Cohort n = 41,787 mother–child pairs	Low Observational	Low levels of caffeine (10–100 mg/day) were associated with significant increases for 10 birth defects High levels (>300 mg/day) had weaker associations, except for craniosynostosis and aortic stenosis. Some of the statistically significant results could be due to chance and should therefore be interpreted with caution	Suggests potential dose-dependent associations between preconception caffeine and specific birth defects. Limited by retrospective design, multiple comparisons, no dose–response, and risk of chance findings. Causality cannot be inferred.
**Coffee consumption & miscarriage** **Nunes et al., 2023** [79]	Prospective Cohort n = 91,462 pregnant women	Moderate Observational	No significant association between maternal coffee consumption and the increased risk of miscarriage	Suggests coffee intake during pregnancy is likely not associated with miscarriage risk. Limited by self-reported intake, inability to assess small/early-pregnancy effects, and potential residual confounding.
**Sugary beverages & pregnancy outcomes Wang et al., 2024** [80]	Prospective Cohort n = 4824 pregnant women	Moderate Observational	Increases risk of GD and gestational hypertension Positive correlation with the occurrence of macrosomia and LGA infants No correlation with miscarriage, LBW, SGA	Indicates frequent sugar-sweetened beverage consumption may contribute to maternal metabolic complications and excess fetal growth. Limited by self-reported intake, potential confounding, and inability to establish causality.
**Dietary salt intake & vascular function Vulin et al., 2024** [81]	Cross-sectional n = 65 pregnant women, 37–40 weeks GA	Low Observational	Salt decreased nitric oxide mediated endothelium-dependent vasodilatation in peripheral micro- and microcirculation No data on oxidative stress outcomes reported. Suggests high salt intake in late pregnancy may impair endothelial function.	Limited by small sample size, cross-sectional design, and reliance on estimated salt intake. Generalizability and causality are limited.
**Maternal caffeine intake & fetal outcomes** **Kukkonen et al., 2024** [82]	Prospective Cohort 1st trimester: n = 2007 3rd trimester: n = 4362	Moderate Observational	Maternal caffeine intake during the last trimester of pregnancy significantly correlated with the caffeine content in the hair of the newborn. The study did not find caffeine consumption to reduce the risk of GD. Moderate to high maternal caffeine intake, even within the current recommendations of under 200 mg/day, was associated with an increased risk of giving birth to an SGA infant, possibly owing to its effects on placental development.	Suggests maternal caffeine, even within current guidelines, may increase SGA risk and is reflected in fetal exposure biomarkers. Limited by self-reported intake, limited mechanistic data, and observational design. Requires replication.
**Maternal macronutrients & maternal-offspring obesity Zhang et al., 2025** [83]	Prospective Cohort n = 66,360 pregnant women	Moderate Observational	Higher maternal protein intake was associated with lower postpartum weight retention and SGA risk, higher LGA and childhood overweight/obesity	Suggests higher maternal protein may have divergent effects: protective for maternal weight/SGA but may increase risk of excess fetal growth and later offspring obesity. Limited by self-reported diet, large-scale observational design, and inability to infer causality or optimal intake levels.
**Maternal tea/coffee consumption & child cognition** **Ouyang et al., 2025** [84]	Prospective Cohort n = 1423 mother–child pairs	Moderate Observational	Infants born to mothers who drank tea throughout pregnancy had higher scores on the assessment of cognitive development compared with those born to mothers who drank tea only in the first trimester. No significant effect of drinking black or green tea on children’s cognitive development up to 36 months of age. Impossibility of accurately identifying the tea ingredient responsible for the results obtained.	Suggests timing and duration of maternal tea intake may be associated with early childhood cognition. Limited by self-reported exposure, observational design, and inability to identify specific tea components. Causal interpretation is not possible.

Legend: n = number; ↑ = increased; ↓ = decreased; → = leads to.

**Table 2:** Influence of consumption of different diets on maternal and offspring health.

Type of Diet	Dietary Patterns, Author	Study Design & Population	Evidence Level	Key Findings	Key Conclusion/Clinical Relevance
**Human studies**					
**Med diet**	Med diet & maternal metabolism/ inflammation Rowley et al., 2022 [85]	Cross-sectional n = 51 pregnant women	Low Observational	Pregnant women exhibit a distinct systemic metabolic profile. Inflammatory biomarkers Glyc A and Glyc B are significantly lower. Positive correlation between BCAA and aromatic amino acids and high Med diet. Increased concentrations of gut-microbial metabolites.	Suggests Med diet in pregnancy may have anti-inflammatory and metabolically favorable effects. Limited by small sample size, cross-sectional design, and lack of clinical outcomes. Causal inference and generalizability are limited.
	Med diet & maternal mental health Flor-Alemany et al., 2022 [14]	Cross-sectional n = 152 pregnant women	Low Observational	Higher adherence to Med diet was associated with better mental health during pregnancy. Positive correlation with emotional regulation, resilience, and positive affect. Negative correlation with anxiety. Adherence to the Med diet is associated with lower levels of pro-inflammatory cytokines, which inhibit brain-derived neurotrophic factor (BDNF), involved in synaptic plasticity and neuronal survival.	Suggests Med diet adherence may support psychological well-being in pregnancy. Limited by small sample size, cross-sectional design, and reliance on self-reported measures. Causal inference is not possible.
	Med diet & offspring overweight/obesity Diaz-López et al., 2024 [88]	Prospective Cohort n = 272 mother–child pairs	Moderate Observational	Adherence to Med diet during pregnancy may protect against the risk of overweight obesity in offspring 4 years old.	Suggests prenatal Med diet may have long-term protective effects against childhood obesity. Limited by small sample size, observational design, and potential for postnatal confounding. Causal inference is limited.
	Med diet & nutritional counseling Coppola et al., 2025 [114]	Intervention study n = 104 pregnant women	Moderate Interventional	Adherence to Med diet during pregnancy may protect the offspring against overweight/obesity at 24 months of age.	Indicates Med diet promotion during pregnancy may reduce early childhood overweight risk. Limited by small sample size, short follow-up, and potential for behavioral co-intervention. Generalizability and causality are limited.
	Med diet & atopic dermatitis Heye et al., 2025 [16]	Prospective Cohort n = 116 mother–child pairs	Moderate Observational	Risk of atopic dermatitis was reduced with consumption of Med diet in pregnancy.	Suggests prenatal Med diet may have protective effects against early childhood allergic skin disease. Limited by small sample size, observational design, and reliance on self-reported diet and outcomes. Causal inference is limited.
	Med diet & food allergy Vassilopoulou et al., 2026 [115]	Prospective Cohort n = 430 mother–offspring dyads	Moderate Observational	Adherence to Med diet with high intake of full-fat dairy products, fruits, and vegetables was associated with reduced risk of food allergy. Higher consumption of poultry, red meat, and fish during pregnancy and breastfeeding may increase this risk.	Indicates specific components within the Med diet may differentially affect offspring allergy risk. Limited by observational design, self-reported exposures/outcomes, and combined pregnancy/lactation exposure window. Causal interpretation is limited.
	Animal studies				
	EVOO phenolic compounds & vertical transmission López-Yerena et al., 2022 [100]	Rat, Lewis strain 3 dietary groups: control, refined olive oil, EVOO n = 8 dams/group, ~20 pups/group	Preclinical Rodent	Significant levels of phenolic compounds and their metabolites in offspring plasma Vertical transmission of EVOO phenolic compounds with health benefits.	Maternal EVOO consumption allows transfer of bioactive phenolics to offspring with potential health benefits. Limited by small sample size, animal model, and lack of functional offspring outcomes. Human translatability is unclear.
	EVOO & maternal immune system/lactation Zhan-Dai et al., 2024 [94]	Rat, Lewis strain 20 female rats EVOO supplementation during pregnancy	Preclinical Rodent	Higher levels of IgA in the mammary glands and breast milk Improve breast milk immune composition.	Maternal EVOO supplementation during pregnancy may enhance passive immunity transfer to offspring via breast milk. Limited by small sample size, animal model, and lack of offspring functional immune outcomes. Human translatability is unclear.
	Med diet vs. WD & maternal microbiota/immunity/metabolism Rio-Aige et al., 2025 [116]	Rat, Wistar dams 3 dietary groups during gestation + lactation 21 days	Preclinical Rodent	Med diet had beneficial impact on mothers compared to WD. Med diet enhanced the mucosal immunity. Med diet influenced the cecal microbiota composition. Med diet increased beneficial taxa of microbiota. Med diet exerts anti-obesogenic effects on lipid metabolism.	Maternal Med diet may improve gut-immune axis and lipid metabolism compared to WD during pregnancy/lactation. Limited by small sample size, animal model, and lack of offspring outcome data. Human translatability is unclear.
**WD**	WD vs. High-fiber diet & offspring metabolic programming Herzl et al., 2023 [117]	Mouse n = 72 dams WD vs. high-fiber unprocessed diet during pregnancy	Preclinical Rodent	WD may influence hyperglycemia and adiposity in offspring. WD determine higher levels of fasting plasma insulin and glucose.	Maternal WD may program offspring metabolic dysfunction. Limited by animal model, small group sizes, and lack of mechanistic data. Human translatability is unclear.
	WD & offspring neurodevelopment Horner et al., 2024 [118]	Observational Cohort n = 593 children	Moderate Observational	WD in pregnancy had a significant association with ADHD and autism.	Prenatal WD exposure may be a risk factor for neurodevelopmental disorders. Limited by observational design, self-reported diet/outcomes, and potential residual confounding. Causal inference is limited.
**Veg diet**	Veg diet vs. Omnivore diet & maternal/neonatal outcomes Przybysz et al., 2023 [119]	Observational Cohort n = 1015 pregnant women	Moderate Observational	The Veg diet did not change the incidence of GD, hypertension and anemia.	Veg diet during pregnancy is not associated with increased risk of key maternal complications. Limited by observational design, heterogeneity of vegetarian patterns, reliance on self-reported diet, and limited outcomes assessed. Generalizability is limited.
	Veg diet vs. Omnivore diet & pregnancy/newborn outcomes Reijonen et al., 2024 [120]	Observational Cohort n = 450: 150 vegetarian, 300 omnivore pregnant women	Moderate Observational	In vegetarian group: The number of SGA newborns was lower; The number of LGA newborns was similar; The birth weight was higher; The number of pregnant women with hypertension did not differ.	Veg diet during pregnancy may be associated with improved fetal growth outcomes without increasing maternal hypertension risk. Limited by observational design, lack of detail on vegetarian subtype and nutrient intake, and potential for residual confounding. Causal interpretation is limited.
**KD**	KD & maternal/fetal development Kosiek et al., 2022 [121]	Rat, Wistar dams n = 30: Normal diet n = 13, KD n = 17 During pregnancy	Preclinical Rodent	KD affects the brain of pregnant females. KD impairs the somatic and neurological development of their offspring. The duration of pregnancy was not affected. A significantly reduced body mass in offspring without morphological anomalies.	Maternal KD during pregnancy may impair offspring growth and neurodevelopment without affecting gestation length. Limited by small sample size, animal model, and lack of mechanistic data. Translation to human pregnancy is limited.
	KD & offspring neuroinflammation Altinöz et al., 2023 [122]	Mouse, C57BL/6 dams n = 12: Standard diet 5% fat n = 5 vs. KD 67.2% fat n = 7 During pregnancy	Preclinical Rodent	Higher brain weight in the offspring. Lower neuronal density. Elevated expression of microglial marker A1F1. Microgliosis. Interfere with the normal development of the embryonic brain NLRP3 and IL-1β expression is higher Maternal weight gain is lower Lower glucose levels compared to the control group, but not hypoglycemia.	Maternal KD during pregnancy may induce neuroinflammatory changes and altered brain development in offspring. Limited by very small sample size, extreme macronutrient composition, animal model, and lack of functional outcomes. Translation to human pregnancy is limited.
	KD & offspring brain biomolecular composition Rugiel et al., 2023 [123]	Rat, Wistar dams KD vs. Normal diet during pregnancy Offspring assessed at 2, 6, 14 days old	Preclinical Rodent	A greater number of abnormalities in brain of the 14 days old offspring like: the increase in the relative level of compounds containing carbonyl groups, the decrease in the relative content of lipids, structural changes in white matter.	Maternal KD during pregnancy may alter offspring brain lipid metabolism and structure. Limited by animal model, lack of functional outcomes, small sample size, and absence of maternal metabolic data. Translation to human pregnancy is limited.
	Partial KD & maternal/offspring outcomes Zala et al., 2025 [112]	Rat, Gestational dams n = 33: Control n = 16 vs. Partial KD n = 17 During pregnancy	Preclinical Rodent	Reduces dam’ litter size and mass. Male offspring reduced lifespan and a late-onset increase in body mass.	Even partial maternal KD during pregnancy may adversely affect reproductive success and offspring metabolic/lifespan outcomes. Limited by small sample size, animal model, lack of mechanistic data, and unclear diet composition. Translation to human pregnancy is limited.

Legend: n = number.

## Data Availability

No new data were created or analyzed in this study. Data sharing is not applicable to this article.

## Abbreviations

The following abbreviations are used in this manuscript:

ABCD	Adolescent Brain Cognitive Development
ADHD	Attention-Deficit/Hyperactivity Disorder
ASD	Autism Spectrum Disorder
BCAA	Branched-chain amino acids
BDNF	Brain-derived neurotrophic factor
BMI	Body mass index
CYP1A2	Cytochrome P450 1A2
DHA	Docosahexaenoic acid
DII	Dietary Inflammatory Index
DNA	Deoxyribonucleic acid
EFSA	European Food Safety Authority
EPA	Eicosapentaenoic acid
EVOO	Extra-virgin olive oil
FAS	Fetal alcohol syndrome
GD	Gestational diabetes
GM	Gut microbiota
HbA1c	Glycated hemoglobin
HPA axis	Hypothalamic–pituitary–adrenal axis
5-HT	5-hydroxytryptamine
IBD	Inflammatory bowel disease
IFN-γ	Interferon gamma
Ig	Immunoglobulin
IL	Interleukin
IR	Insulin resistance
IUGR	Intrauterine growth restriction
KD	Ketogenic diet
LBW	Low birth weight
LCPUFA	Long-chain polyunsaturated fatty acids
LGA	Large for gestational age
MAC	Microbiota-accessible carbohydrates
MedDiet	Mediterranean diet
mRNA	Messenger RNA
MUFA	Monounsaturated fatty acids
NBDPS	National Birth Defects Prevention Study
POMC	Pro-opiomelanocortin
PUFA	Polyunsaturated fatty acids
RNA	Ribonucleic acid
ROS	Reactive oxygen species
SCFAs	Short-chain fatty acids
SGA	Small for gestational age
Th	T helper cells
TL	Telomere length
Treg cells	Regulatory T cells
WD	Western diet
